# VvmiR160s/*VvARFs* interaction and their spatio-temporal expression/cleavage products during GA-induced grape parthenocarpy

**DOI:** 10.1186/s12870-019-1719-9

**Published:** 2019-03-21

**Authors:** Wenying Zhang, Mostafa Abdelrahman, Songtao Jiu, Le Guan, Jian Han, Ting Zheng, Haifeng Jia, Changnian Song, Jinggui Fang, Chen Wang

**Affiliations:** 1 College of Horticulture, Nanjing Agricultural University, Nanjing, 210095 China; 2 Department of Botany, Faculty of Sciences, Aswan University, Aswan, 81528 Egypt; 3 Arid Land Research Center, Tottori University, Tottori, 680-001 Japan; 4 Department of Plant Science, School of Agriculture and Biology, Shanghai Jiao Tong University, Shanghai, China

**Keywords:** VvmiR160s, *VvARFs*, Grape, Flower, Gibberellin, Parthenocarpy

## Abstract

**Background:**

Grape (*Vitis vinifera*) is highly sensitive to gibberellin (GA), which effectively induce grape parthenocarpy. Studies showed that miR160s and their target *AUXIN RESPONSIVE FACTOR* (*ARF*) responding hormones are indispensable for various aspects of plant growth and development, but their functions in GA-induced grape parthenocarpy remain elusive.

**Results:**

In this study, the morphological changes during flower development in response to GA treatments were examined in the ‘Rosario Bianco’ cultivar. The precise sequences of *VvmiR160a/b/c/d/e* and their *VvARF10/16/17* target genes were cloned, sequenced and characterized. The phylogenetic relationship and intron-exon structure of VvARFs and other ARF family members derived from different species were investigated. All VvmiR160s (except VvmiR160b) and *VvARF10/16/17* had the common *cis*-elements responsive to GA, which support their function in GA-mediated grape parthenocarpy. The cleavage role of VvmiR160s-mediated *VvARF10/16/17* was verified in grape flowers. Moreover, spatio-temporal expression analysis demonstrated that among *VvmiR160* family, *VvmiR160a/b/c* highly expressed at late stage of flower/berry development, while *VvARF10/16/17*showed a reverse expression trend. Interestingly, GA exhibited a long-term effect through inducing the expression of *VvmiR160a/b/c/*e to increase their cleavage product accumulations from 5 to 9 days after treatment, but GA enhanced the expressions of *VvARF10/16/17* only at short term. Pearson correlation analysis based on expression data revealed a negative correlation between VvmiR160a/b/c and *VvARF10/16/17* in flowers not berries during GA-induced grape parthenocarpy.

**Conclusions:**

This work demonstrated that the negative regulation of *VvARF10/16/17* expression by *VvmiR160a/b/c* as key regulatory factors is critical for GA-mediated grape parthenocarpy, and provide significant implications for molecular breeding of high-quality seedless berry.

**Electronic supplementary material:**

The online version of this article (10.1186/s12870-019-1719-9) contains supplementary material, which is available to authorized users.

## Background

In angiosperms, flowering-time is a crucial part of the plant life cycle, providing a critical developmental switch from the vegetative growth to the reproductive stage to ensure seed production required for the survival of plant [1]. Floral development and flowering-time is regulated by a complex networks of genetic and epigenetic reprogramming [photoperiod, autonomous, vernalization/temperature, gibberellin (GA), and sucrose pathways] to ensure that flowering-time has coincided with suitable conditions for fertilization and seed dispersal [2–7]. This genetic and epigenetic reprogramming is attained by explicit control of the expression of key flowering genes at both transcriptional and post-transcriptional level in response to developmental and environmental stimuli [3, 4]. In this context, microRNAs (miRNAs), a class of small, single-stranded, non-coding RNAs ranging from 19 to 25 nucleotides (nt) in length, have recently been identified as crucial regulators of gene expression through transcript cleavage and translational inhibition of target genes involved in the floral transition, floral patterning and development of floral organs [8–15]. However, the biological functions of these miRNAs have been characterized mainly in model plants [16, 17], while their roles during grapevine (*Vitis vinifera*) parthenocarpy and early ripening processes are still not fully understood.

Phytohormones, such as GA, are known to be essential for the regulation of grape flower development, berry expansion, ripening, and seedless berry induction, and the exogenous application of GA can trigger fruit parthenocarpy and early ripening in various grapevine cultivars [15, 18, 19]. Several reports demonstrated that different miRNA families (miR061, miR156, miR159, miR160, miR164, miR167, miR172, and miR319) and their target genes are implicated in GA signal during floral transition and fruit set development [8, 9, 20]. For instance, *Arabidopsis* AtmiR159 acts as a homeostatic regulator of GA signaling to direct the cleavage of genes encoding AtGAMYB proteins, causing a defect in anthers development and a subsequent reduction in floral fertility [21]. Similarly, auxin (AUX) is also known to be essential for fruit development and repining, and AUXIN RESPONSE FACTORs (ARFs) regulate the expression of a large set of auxin-responsive genes by binding to AUXIN RESPONSEELEMENTs (AuxREs) in their promoters [19]. Several studies in *Arabidopsis* and tomato plants demonstrated that miRNAs play significant roles in AUX signaling through controlling transcript abundance of *ARF2/3/4/6/8/10/16* and *17* [22–24]. For example, *Arabidopsis miR160a (AtmiR160a)* mutant plants exhibited a significant reduction in *AtmiR160a* expression in *floral organs in carpel* (*foc*) inflorescences and irregular flower phenotype due to various embryonic defects [17]. Besides these, *AtmiR160a* mutant plants displayed up-regulation in *ARF16* and *ARF17* during embryo development in *foc* plants relative to wild-type plants, suggesting that *AtmiR160a* is essential for embryo development in *Arabidopsis* through negative regulation of *ARF* genes [17]. Similarly, tomato (*Solanum lycopersicum*) *SlmiR160* knocked down plants exhibited a perturbed ovary patterning and abnormal floral organ abscission, and the down-regulation of *SlmiR160* was associated with the up-regulation of *SlARF10A*, *SlARF10B*, and *SlARF17*, suggesting that SlmiRNA160 is needed for AUX-mediated floral and fruit development [24]. Although *ARFs* appear to have unique functions in some contexts, they also display overlapping functions among different plant species, for instance, a mutation in *ARF8* resulted in the formation of seedless fruit in *Arabidopsis* and tomato plants [25, 26]. Therefore, characterization of *ARF* members and their roles in floral development in different plant species are crucial.

Over the last decades, there has been a considerable increase in the economic importance of grape production for direct berry consumption and beverage production [27]. There are several reports demonstrated that the developmental process of grape flower affects berry fruiting type, yield and quality [28]. Therefore, the molecular regulatory mechanisms of grape flower development are essential to be determined. In our recent study, grapevine berries-treated with GA_3_ exhibited a significant up-regulation in several *miRNA* families in floral tissues, including *miR159s* and *miR160s*, suggesting a potential role of these miRNAs in GA signaling [29]. The potential role of VvmiR159c in GA signaling through GA-DELLA [SLENDER RICE 1 (SLR1)]-VvmiR159c-*VvGAMYB* as a key molecular module for seedless grapevine development has recently been reported by our group [15]. However, the molecular mechanisms of miR160 and their specific target genes on modulating grape flower and parthenocarpy development remain elusive. In this context, we first examined the morphological changes in ‘Rosario Bianco’ cultivar during flower and berry development in response to GA_3_ treatment. Secondly, we isolated and cloned the precise sequences of *VvmiR160a/b/c/d/e* in the floral tissues of grape cv. ‘Rosario Bianco’. Thirdly, their *ARF* target genes were predicted by using *VvmiR160a/b/c/d/e* sequences, together with updated grape mRNA database. The identified VvARFs were further characterized for their phylogenetic relationship and sequence homology with other ARFs derived from various plant species. The promoter motifs responsive to hormones including GA in*VvMIR160s* (VvmiR160 precursors) and *VvARFs* were analyzed, which was confirmed during our experiments. Finally, the cleavage role and cleavage sites of the different *VvmiR160* members and their *VvARF10/16/17*target genes were verified in grape flowers by RNA-ligase-mediated Rapid Amplification of cDNA Ends (RLM-RACE) and Poly (A) polymerase -mediated 3′ rapid amplification of cDNA ends (PPM-RACE). In addition, the temporal-spatio expression and cleavage product accumulation of *VvmiR160a/b/c/d/e* and *VvARF10/16/17* target genes in response to GA application during grape development were investigated. Our results demonstrated that VvmiR160 modulates GA-mediated floral developmental and grapevine parthenocarpic regulation via its negative regulation of *VvARF* activity.

## Results

### Morphological changes of the floral and berry development during grapevine parthenocarpic process-induced by exogenous GA treatment

**Fig. 1:**
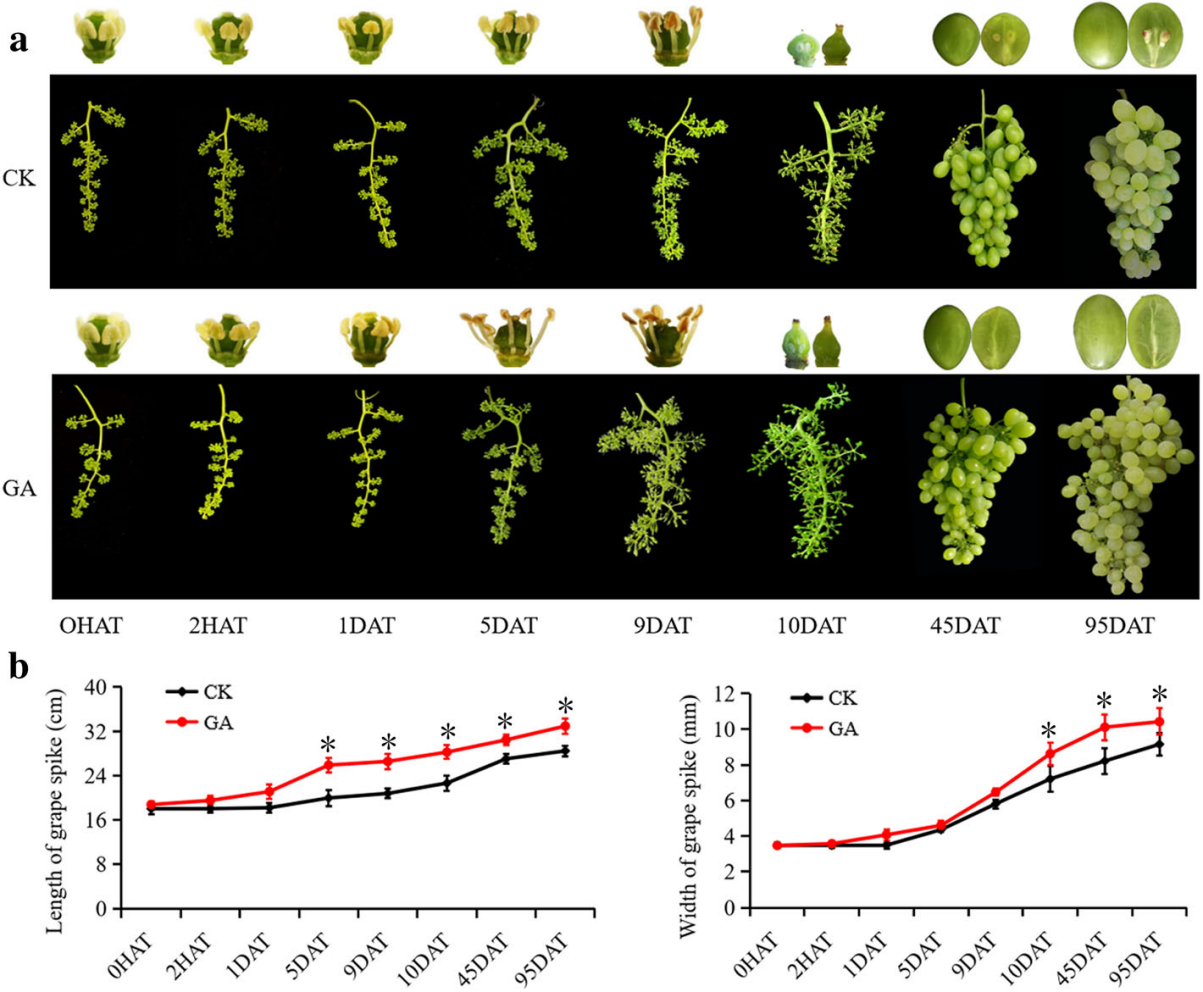
Morphological changes of the floral and berry development during grapevine parthenocarpy process-induced by exogenous gibberellin (GA) application. **a** Morphological changes of ‘Rosario Bianco’ cultivar during flower and berry development in response to GA_3_ treatment at different time points [0 h, 2 h, 1 day, 5 , 9 , 10 , 45 and 95 days after treatment (0HAT, 2HAT, 1DAT, 5DAT, 9DAT, 10DAT, 45DAT and 95DAT, respectively)]. **b** Effect of GA_3_ on the length and width of grape spikes. Values are means ± standard errors (SEs) of three independent biological replicates (*n* = 10). Asterisks indicate a significant difference between GA-treated plants and respective untreated control (CK) plants at each time point as determined by Student’s *t*-test (*P < 0.05; **P < 0.01)

To investigate the effect of GA_3_ on grapevine floral development and parthenocarpy process, we compared the floral clusters and berries morphology of GA_3_-treated ‘Rosario Bianco’ grapevine cultivar relative to untreated control plants (CK) at different time points [0 h, 2 h, 1 , 5 , 9 , 10 , 45 and 95 days after treatment (0HAT, 2HAT, 1DAT, 5DAT, 9DAT, 10DAT, 45DAT and 95DAT respectively)] (Fig. 1a and b). Out of them, GA treatment was performed at 21 days after inflorescence, and the GA-treated plants reached anthesis at 9 DAT, while the control plants did not. As shown in Fig. 1a and b, the GA_3_-treated plants exhibited a significant early anthesis and a remarkable increase in the length of spikes at 5DAT and 9DAT in comparison with untreated control plants (Fig. 1b). The GA_3_-treated inflorescences start opening on the 5DAT, while the control inflorescences remained closed (Fig. 1a). After peeling off buds for further observation, an obvious effect of GA_3_ treatment on ‘Rosario Bianco’ grapevine inflorescence was strongly observed on 5DAT and 9DAT with distinct floral morphology, including dark yellow and small anthers, long filaments, short styluses and big ovaries relative to untreated control plants (Fig. 1a). In addition, GA_3_-treated grapevine berries exhibited the long berry spikes and grains in contrast with control ones (Fig. 1b), and the high seedless ratio (96.2%) relative to control plants (~ 5%) (Fig. 1a), suggesting that GA_3_ treatment possessed the significant effect in inducing ‘Rosario Bianco’ grapevine parthenocarpy.

### Determination of the precise sequences of VvmiR160s in the floral tissues of grape cv. ‘Rosario Bianco’ by miR-RACE

**Fig. 2:**
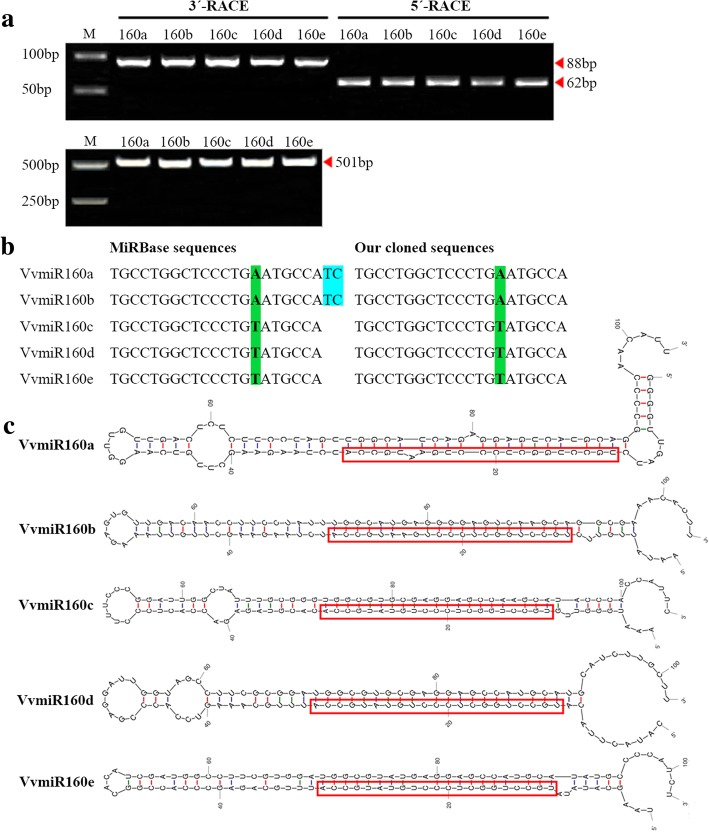
Mature sequences of VvmiR160s and the secondary structures of their precursors. **a** The PCR products of 3′-miR-RACE and 5′-miR-RACE for VvmiR160a/b/c/d/e, respectively, and the cloning of their primary sequences. **b** Comparison of our cloned VvmiR160s precise sequences with the homologous sequences in grapes from MiRBase. **c** The secondary structures of VvmiR160s precursors, where red frames indicate the mature sequences of VvmiR160s at the 5′-end arms of their precursor structures

In this study, we employed miR-RACE technology to determine the precise sequences of VvmiR160 family members in the floral tissues of grapevine cv. ‘Rosario Bianco’ (Fig. 2a and b). The PCR products of 3′- and 5′-miR-RACE for VvmiR160a/b/c/d/e members were ~ 88 and 62 bp, respectively (Fig. 2a). All the miR-RACE PCR products were validated by subsequent cloning and sequencing. The sequence identity between our cloned sequences and validated miRNA in miRBase 21.0 was used to confirm the precision of the 3′- and 5′-miR-RACE technique. *VvmiR160a* and *VvmiR160b* were identical both in length (21 nt) and nucleotide sequence “TGCCTGGCTCCCTGAATGCCA”, with 14.2; 23.8; 23.8 and 38.0 percentage composition of A, T, G, and C, respectively (Fig. 2b). Whereas, *VvmiR160c*, *VvmiR160d* and *VvmiR160e* shared identical length and sequence “TGCCTGGCTCCCTGTATGCCA”, with 9,5; 28,5; 23,8 and 38,0 percentage composition of A, T, G and C, respectively (Fig. 2b). The more GC content in the VvmiR160s is important for stabilization of the stem-loop hairpin structure. Both VvmiR160a/b and VvmiR160c/d/e exhibited high sequence similarity with one base variation (A/T) at the 15th site from the 5′-end (Fig. 2b). The primary transcripts of VvmiR160a/b/c/d/e (*VvMIR160a-e*) were cloned and sequenced, demonstrating that their lengths were about 501 bp (Fig. 2a), which were further used for secondary hairpin structure formation using Mfold online tool (http://unafold.rna.albany.edu/). These results confirmed the authenticity of VvmiR160a/b/c/d/e, and all VvmiR160a/b/c/d/e members were located in the 5′-stem arm of their precursor’s loop-stem structures (Fig. 2c), suggesting that various members of VvmiR160 family possess a conserved position at their precursors.

### Identification, characterization and chromosomal distribution of *VvARF* genes

**Fig. 3:**
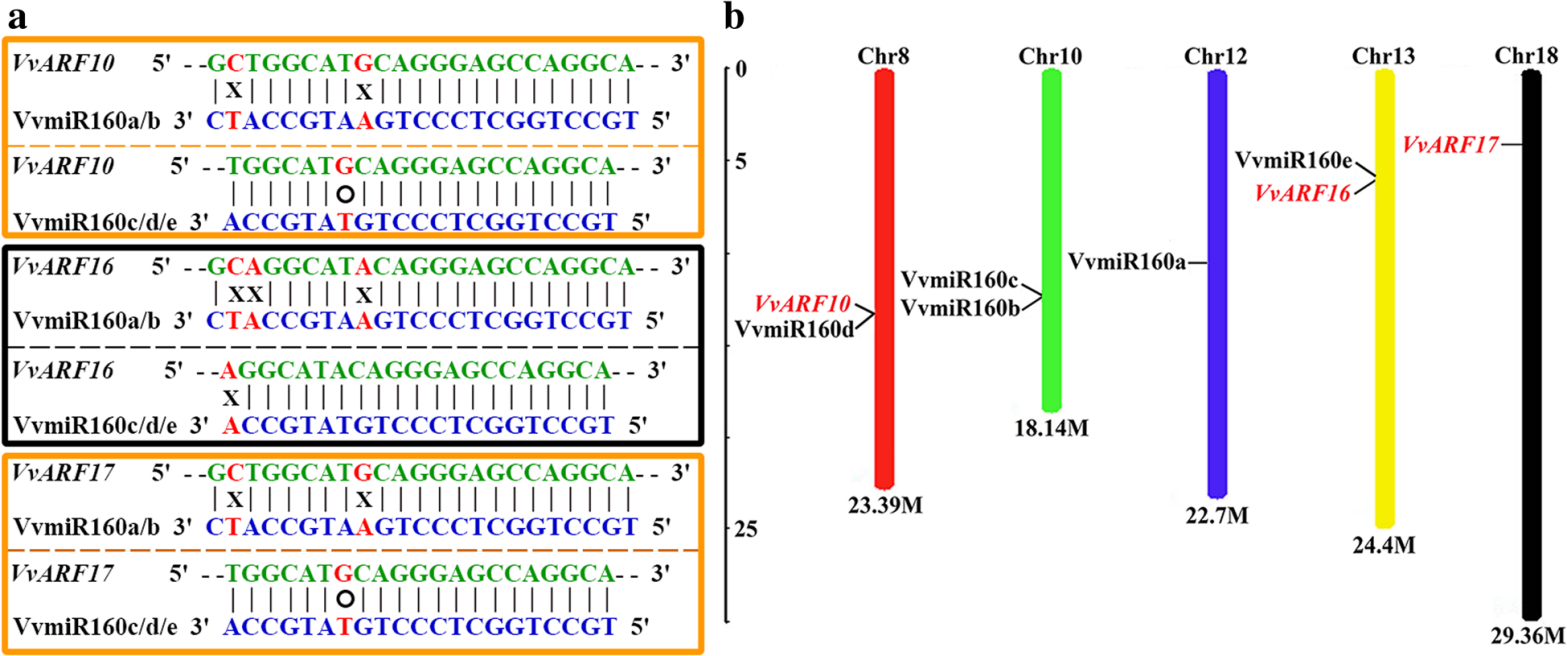
Complementary degree and distribution of VvmiR160s and their *VvARF* targeted genes on grapevine chromosomes. **a** The complementary degree of VvmiR160s and their potential *VvARF* target genes. ‘X’ represents complementary mismatch, and ‘O’ represents the 0.5 mismatches. **b** The distribution of VvmiR160s and *VvARFs* on grapevine chromosomes. The chromosome numbers and sizes (Mb) are indicated at the top and bottom of each bar, respectively

Based on our identified sequences of VvmiR160s and the updated grape mRNA database (GRAPE_IGGP 12X assembly - v2 annotation), we employed the psRNATarget software (http://plantgrn.noble.org/psRNATarget/#) to re-predict the specific target genes of VvmiR160s following the rules of target prediction as reported by Wang [30]. *VvARF10*/*16*/*17* were identified as the candidate target genes of *VvmiR160s* (Additional file 1: Figure S1), and all target regions of VvmiR160s occurred in the coding sequence (CDS) of the corresponding *VvARF* target genes, and their interaction modes were cleaved (Table 1). Next, the complementary degree of *VvmiR160*:*VvARF* pairs exhibited some divergence, for example,*VvmiR160a/b* and their *VvARF16* target genes have three mismatch bases, while *VvmiR160c/d/e* displayed only one mismatch base with *VvARF16* (Fig. 3a), indicating some divergence in their cleavage roles. Also, high conservation at the sites with mismatch bases between VvmiR160s and *VvARFs* was observed (Fig. 3a), which might be one of the reasons for the conservation of target modes for VvmiR160s. All VvmiR160s and their *VvARF* target gene sequences were further mapped in grapevine chromosomes (Chrs) using MapInspect software (http://www.plantbreeding.wur.nl/uk/software-mapinspect.html). All five *VvmiR160s* and three *VvARFs* identified in this study were distributed over four of the 19 grapevine chromosomes (Fig. 3b). Chr 10 contained two VvmiR160 members, whereas Chr 8, 12 and 13 contained only one VvmiR160 member (Fig. 3b). Mapping results demonstrated that VvmiR160a, VvmiR160d, and VvmiR160e are located on Chr 12, 8, 13 respectively, while both VvmiR160b and VvmiR160c were mapped into the same position on Chr 10 (Fig. 3b). In addition, *VvARF10*, *VvARF16*, and *VvARF17* were mapped on Chr 8, 13 and 18, respectively (Fig. 3b). VvmiR160d:*VvARF10* and VvmiR160e:*VvARF16* pairs were located in the same position on Chr 8 and 13, respectively, whereas there is no co-localization of miR160s with *VvARF17* on Chr 18 (Fig. 3b). Whether this chromosome neighboring of *VvmiR160s* and *VvARFs* might affect their cleavage activity, this needs further investigation.

**Table 1:** Prediction of VvmiR160 target genes by bioinformatics

MiR-Acc.	MiRNA mature sequence	MiRNA length	Target_Acc.	Expectation	UPE	Target regions	Inhibition	Multiplicity
VvmiR160a/b	TGCCTGGCTCCCTGAATGCCATC	23	VIT_218s0001g04180.1 (*VvARF17*)	1	20.766	CDS:1755–1777	Cleavage	1
		23	VIT_208s0040g01810.1 (*VvARF10*)	1	23.188	CDS:2389–2411	Cleavage	1
		23	VIT_208s0040g01810.3 (*VvARF10*)	1	23.188	CDS:2513–2535	Cleavage	1
		23	VIT_208s0040g01810.2 (*VvARF10*)	1	23.188	CDS:2389–2411	Cleavage	1
		23	VIT_208s0040g01810.4 (*VvARF10*)	1	23.188	CDS:2534–2556	Cleavage	1
		23	VIT_218s0001g04180.2 (*VvARF17*)	1	20.766	CDS:1755–1777	Cleavage	1
		23	VIT_213s0019g04380.1 (*VvARF16*)	1	22.721	CDS:1566–1585	Cleavage	1
		23	VIT_213s0019g04380.3 (*VvARF16*)	1	22.721	CDS:1566–1585	Cleavage	1
		23	VIT_213s0019g04380.4 (*VvARF16*)	1	22.721	CDS:1701–1720	Cleavage	1
		23	VIT_213s0019g04380.2 (*VvARF16*)	1	22.721	CDS:2147–2166	Cleavage	1
VvmiR160c/d/e	TGCCTGGCTCCCTGTATGCCA	21	VIT_218s0001g04180.1 (*VvARF17*)	0.5	20.766	CDS:1757–1777	Cleavage	1
		21	VIT_208s0040g01810.1 (*VvARF10*)	0.5	23.188	CDS:2391–2411	Cleavage	1
		21	VIT_208s0040g01810.3 (*VvARF10*)	0.5	23.188	CDS:2515–2535	Cleavage	1
		21	VIT_208s0040g01810.2 (*VvARF10*)	0.5	23.188	CDS:2391–2411	Cleavage	1
		21	VIT_208s0040g01810.4 (*VvARF10*)	0.5	23.188	CDS:2536–2556	Cleavage	1
		21	VIT_218s0001g04180.2 (*VvARF17*)	0.5	20.766	CDS:1757–1777	Cleavage	1
		21	VIT_213s0019g04380.1 (*VvARF16*)	0	22.721	CDS:1566–1585	Cleavage	1
		21	VIT_213s0019g04380.3 (*VvARF16*)	0	22.721	CDS:1566–1585	Cleavage	1
		21	VIT_213s0019g04380.4 (*VvARF16*)	0	22.721	CDS:1701–1720	Cleavage	1
		21	VIT_213s0019g04380.2 (*VvARF16*)	0	22.721	CDS:2147–2166	Cleavage	1

Note: The targeted information of VvmiR160s and *VvARFs* obtained by Pstarget software (http://plantgrn.noble.org/psRNATarget/#)

### Identification of *VvARFs* and phylogenetic and diversified analyses of their orthologous sequences across diverse plant species

**Fig. 4:**
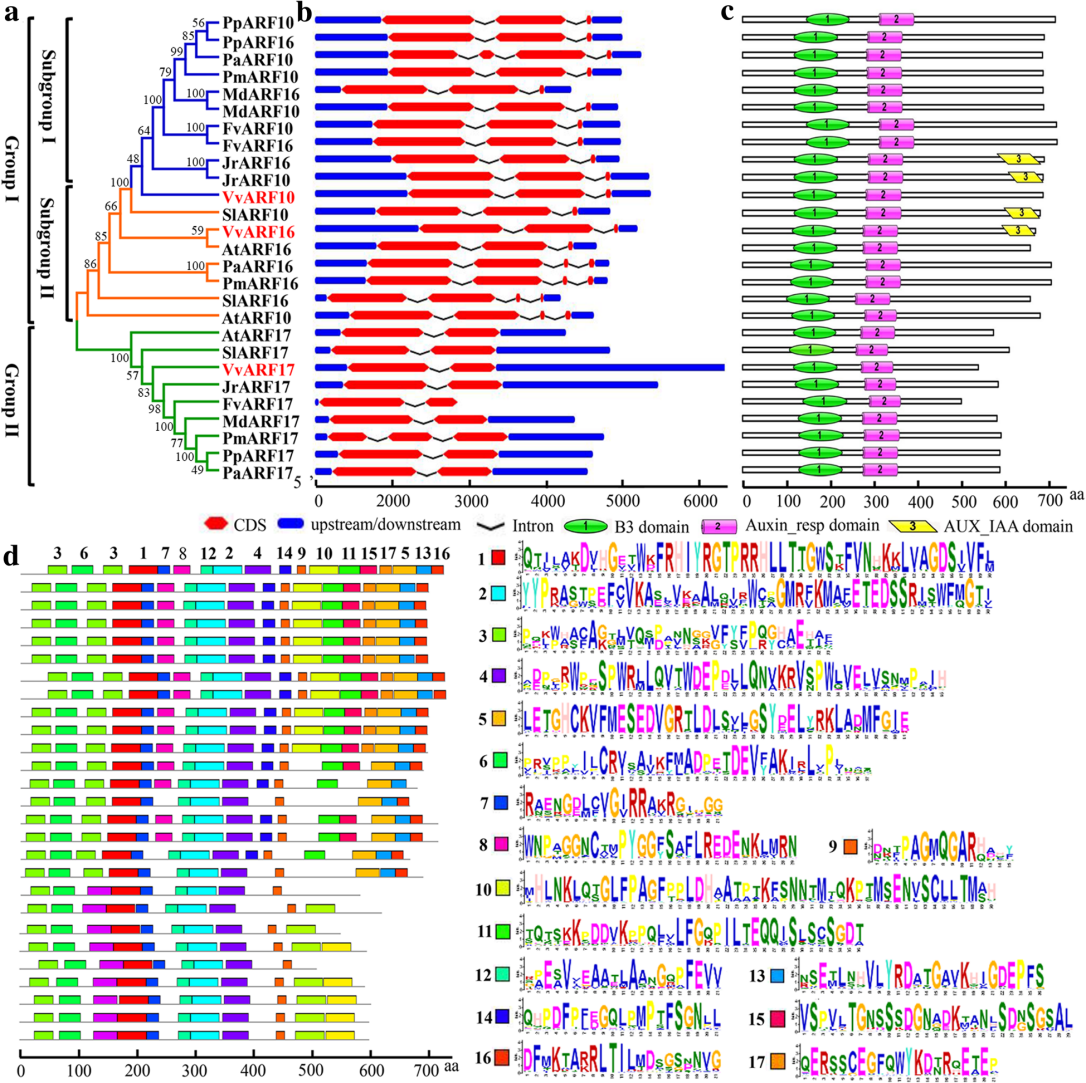
The phylogenetic relationship, exon-intron organization, and conserved domain analyses among the grapevine ARF proteins and their orthologous sequences across different plant species. **a** The unrooted tree was generated using MEGA 7.0.21 program by neighbor-joining method. Bootstrap values from 1000 replicates are indicated at each branch. **b** The exon-intron composition of *ARF* genes. The coding (CDS) and up−/down-stream regions are represented by red and blue boxes, respectively. Lines represent the introns. **c** Conserved domains in ARF proteins determined by searching the ARF protein sequences in NCBI Conserved Domain Database. B3: plant-specific B3-DNA binding domain, Auxin_resp: a conserved region of an auxin-responsive factor, AUX_IAA: C-terminal AUX_IAA domain. **d** The conserved motifs of ARF proteins in nine species were performed using the MEME program and arranged corresponding to the phylogenetic tree. Different motifs are highlighted with different color boxes and numbers. The length of boxes corresponded to motif length

We cloned the three predicted *VvARF10/16/17* gene sequences in ‘Rosario Bianco’ grapevine flowers, and their deduced amino acid sequences were identified (Additional file 2: Figure S2). The open reading frame (ORF) of *VvARF10* was 2106 bp encoding 701 amino acid residues, *VvARF16* exhibited 2052 bp encoding 683 amino acid residues, while *VvARF17* possessed 1653 bp encoding 550 amino acid residues (Additional file 2: Figure S2). To study the phylogenetic relationships between the members of ARF family, an unrooted tree was constructed from an alignment of protein sequences of VvARF10/16/17 and their orthologous from different plant species by using Neighbour-joining method (Fig. 4a). Phylogenetic analysis reveals diversification and conservation of ARFs among different plant species (Fig. 4a). All the ARF family members fell into two major groups represented by 27 accessions (Fig. 4a). Group I contained all ARF10 and ARF16 classes, whereas group II contained all ARF17 class (Fig. 4a). The phylogenetic relationship between our VvARFs and orthologues one derived from NCBI demonstrated that VvARF16 has a close phylogenetic relationship with *Arabidopsis* (AtARF16), while VvARF10 and VvARF17 exhibited the closest relation to walnut (*Juglans regia*, JrARF10, and JrARF17, respectively), indicating a conserved nature of ARF family members among diverse plant species. On the other hand, VvARF10/16/17 have a distance phylogenetic relationship with cherry (*Prunus avium*, PaARFs) and peach (*Prunus persica*, PpARFs), indicating the diversification of evolution in diverse members of ARF protein family (Fig. 4a).

Analysis of the exon-intron structure of the three *VvARF* full-length cDNA sequences with the corresponding genomic DNA sequences showed that several members of *VvARF*-CDS are disrupted by one to three introns (Fig. 4b). The maximum number of introns was detected in *ARF10/16* members, while the lowest was detected in *ARF17* (Fig. 4b). All the *ARF* members exhibited similar gene structures of intron position and length similar to their phylogenetic tree, which supports the reliability of the phylogenetic analysis (Fig. 4b). Three conserved domains (B3, AUX_rsep, and AUX_IAA) were identified, and their distribution in the proteins of respective ARFs in the phylogenetic tree has been presented (Fig. 4c). All the ARF proteins contained two well-conserved DNA binding domains B3 and Auxin_resp at the N-terminus (Fig. 4c). While VvARF16, SlARF10, JrARF10, and JrARF16 had additional AUX_IAA domain in the C-terminus (Fig. 4c), suggesting that ARFs have functional conservation in response to AUX signal in various plant species.

Next, motif composition analysis of ARFs proteins was carried out using the MEME 5.05 tool. The homologous ARFs with more close relationships from the phylogenetic tree possessed the similar motif compositions, and those with more far relationships had the conspicuous difference in their motif compositions (Fig. 4d), implying the more similar structures of homologous ARFs, the more conservation of their functions. Furthermore, the parameters of protein physicochemical properties, including the number of amino acids, the molecular weight, the isoelectric point, the aliphatic index and the grand average of hydropathicity were analyzed (Additional file 3: Table S1). In contrast with the results in Fig. 4a and d, we revealed that although VvARF10 and JrARF10; VvARF16 and AtARF16; VvARF17 and JrARF17, had the close genetic distance in their gene sequences, their parameters in proteins had much more variation. This range of variability indicates that different ARF proteins might operate in various microenvironments [31].

### Cis-element analysis of promoters of *VvMIR160s* and their *VvARF* target genes

**Fig. 5:**
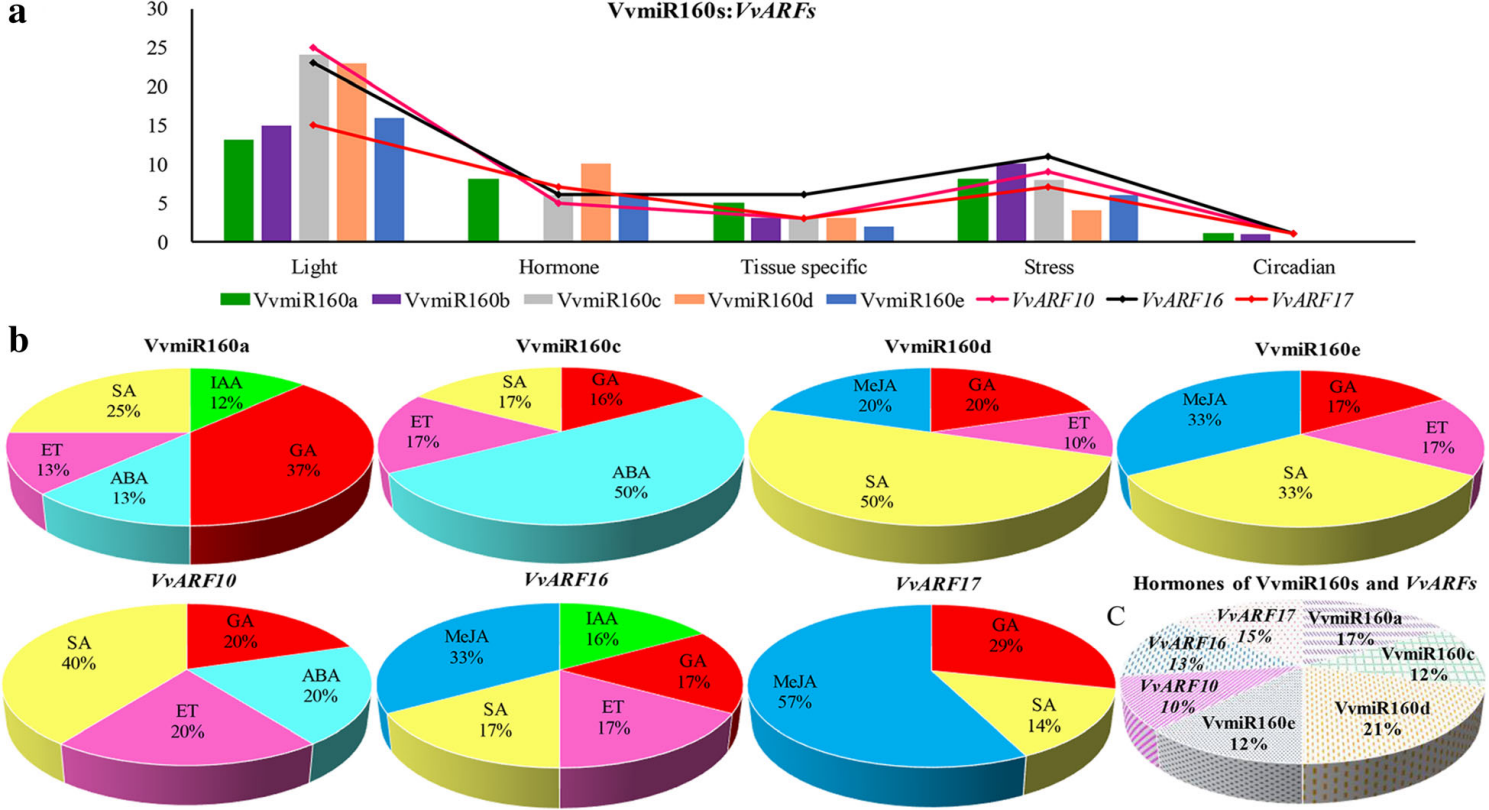
Motif analysis of the promoters from the *VvMIR160s* precursor and their target *VvARF* genes. **a** The total number of diverse types of motifs derived from *VvMIR160s* and *VvARFs* promoters which are involved in the different biological process. **b** The proportion of each type of hormone-related elements of *VvMIR160s* and its *VvARF* targeted genes respectively. **c** The percentage of the total amount of the hormone-related *cis*-elements of *VvMIR160s* and its *VvARF* targeted genes respectively

The promoter region is a critical factor for gene expression, and its sequence contains some essential *cis*-elements (functional components) which could reflect the potential functions of genes [32]. To recognize the possiblefunctions of *VvMIR160s* and their *VvARFs* target genes in grapes, we analyzed the *cis*-elements in their promoters (Additional file 4: Table S2). All *cis*-elements were classified into five types, including light, hormone, tissue-specific, circadian and stress responsive elements (Fig. 5a). Light-related elements were the most abundant among all five types of motifs in the different VvmiR160 and *VvARF* members (Fig. 5a), which might be due to the important role of light in the photosynthesis process for plant growth and development. Whereas, the *cis*-elements responsive stress, hormone, and specific tissues development were also detected; however, their abundance was much lower than those of light responsive *cis*-element (Fig. 5a). Moreover, there were diverse in the type and number of promoters’ *cis*-elements of *VvMIR160s* and their target *VvARFs* (Fig. 5a and Additional file 4: Table S2), indicating the diversification of various members from the same one family.

To further recognize the possible regulation mechanism of *VvmiR160s* and their *VvARF* target genes in response to GA treatmentduring grape flower development, we scanned the hormone-related *cis*-elements in their promoter regions (Additional file 5: Table S3). Interestingly, we found that almost all VvmiR160 members (except VvmiR160b) and their *VvARF10/16/17* target genes, had hormone responsive *cis*-elements, including indole acetic acid (IAA), GA, methyl jasmonate (MeJA), salicylic acid (SA), abscisic acid (ABA) and ethylene (ET) responsive *cis*-elements (Fig. 5b). However, both the composition and quantity of *cis*-elements related to hormones exhibited some variance among different VvmiR160 members and their *VvARF* target genes (Fig. 5b and c), indicating that their potential functions might possess some unique as well as overlapping functions in grapes. Interestingly, all VvmiR160s (except VvmiR160b) and *VvARFs* had the common *cis*-elements responsive to GA and SA, which could be essential hormones for modulating grape flower and berry development. For example, VvmiR160a/c/d/e have *cis*-elements responsive to GA, among which, VvmiR160a exhibited the highest percentage (37%), while VvmiR160c displayed the lowest (16%). Similarly, VvmiR160d and VvmiR160e exhibited the highest percentage of *cis*-elements responsive to SA (50 and 33%, respectively) in comparison with VvmiR160a and VvmiR160c (Fig. 5b). Our results supported the significant information for the prediction of the potential functions of VvmiR160:*VvARF* pairs in the modulation of grape growth and development through responding hormone signals.

### In vitro activity of *VvMIR160c* and *VvARF10* promoters in response to GA

**Fig. 6:**
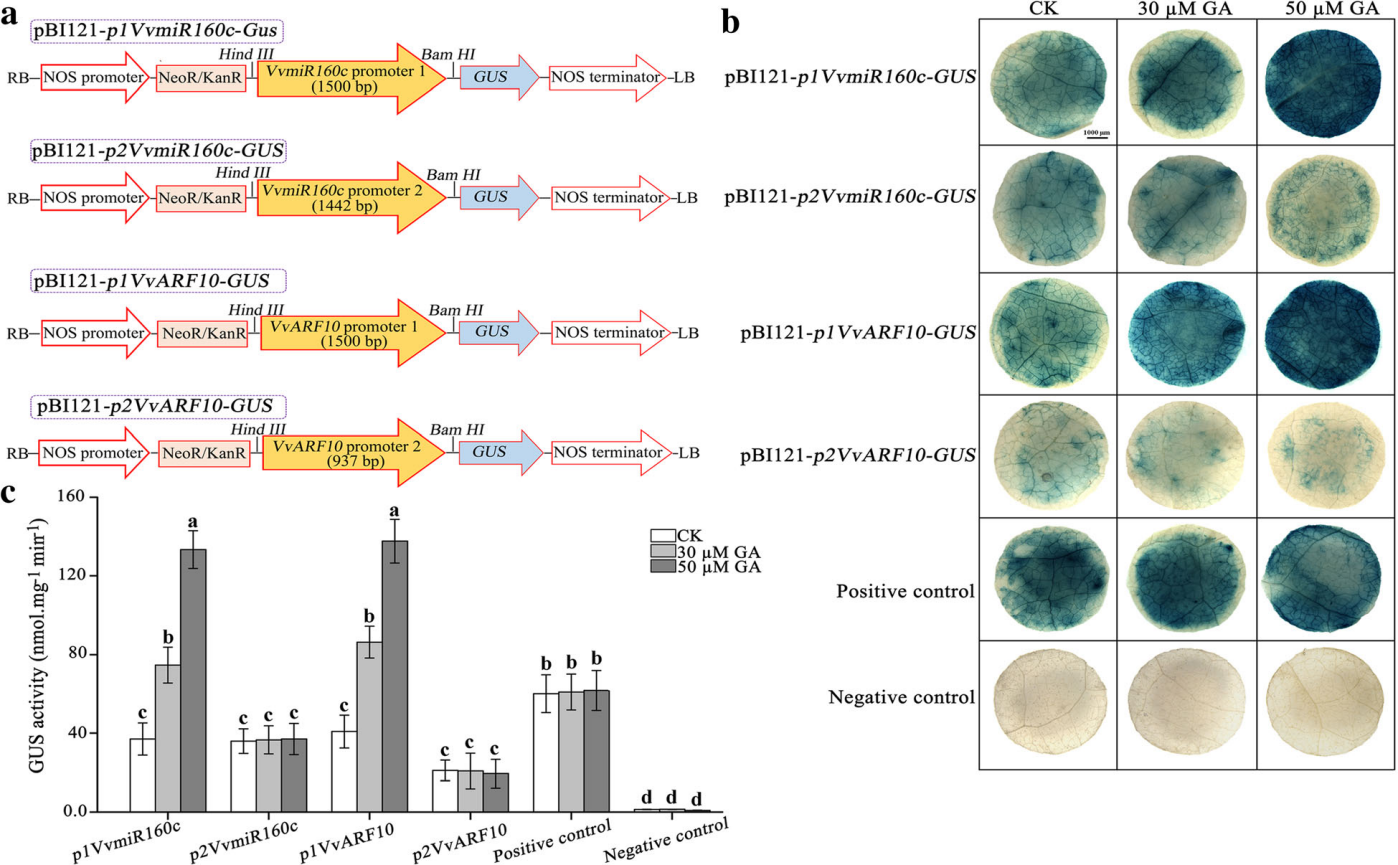
Functional analyses of the *VvMIR160c* and *VvARF10* promoters. **a** Schematic diagram of the pBI121-*pVvmiR160c/VvARF10-GUS* constructs. pBI121-*p1VvMIR160c-GUS* and pBI121-*p1VvARF10-GUS* constructs. The 1.5 kb promoter regions of *VvMIR160c* and *VvARF10* were cloned into pBI121 to replace the *35SCaMV* promoter and were used to drive the *GUS* gene. pBI121-*p2VvMIR160c-GUS* constructs. The 1442 bp promoter fragment region of *VvMIR160c* without including the GA response element (59–1500 bp) was cloned into pBI121 to replace the *35SCaMV* promoter and was used to drive the *GUS* gene. pBI121-*p2VvARF10-GUS* constructs. The 937 bp promoter fragment region of VvmiR160c without including the GA response element (564–1500 bp) was cloned into pBI121 to replace the *35SCaMV* promoter and was used to drive the *GUS* gene. **b** and **c** Histochemical staining pattern (**b**) and GUS activity (**c**) of tobacco leaves in response to different treatments. The fully expanded leaves from 6-week-old tissue culture plants of tobacco were infiltrated with Agrobacterium carrying different constructs. The infiltrated seedlings were then moved back to the environmental chamber and kept in the dark for 3 d. Treatment on tobacco leaves with 30 μM GA and 50 μM GA were performed 2 days later. The uniformly sized leaves were used in infiltration experiment. The presented images of leaves in (**b**) are representative of the results obtained from three independent experiments. Data shown in (**c**) represent the mean ± SD of three independent experiments (*n* = 3). Different letters denote significant differences between the constructs and the treatments (*P* < 0.05), positive control (pBI121); negative control (pBI101)

A further analysis of the *VvMIR160c* and *VvARF10* promoters were conducted in order to confirm the activity of their promoters in response to GA. The *VvMIR160c* and *VvARF10* promoter regions were fused to the *β-glucuronidase (GUS)* reporter gene after removal of *35SCaMV* promoter in the binary vector pBI121, and the vector constructs are shown in Fig. 6a. The obtained constructs were transformed independently into tobacco plants using the *Agrobacterium*-mediated method. A pBI121-*35S-GUS* construct-containing *35SCaMV* promoter served as positive control, while pBI101 construct-lacking *35SCaMV* promoter served as negative control (Fig. 6b). To validate the presence of GA-related *cis*-elements in *VvMIR160c* and *VvARF10* promoter regions, 0, 30 and 50 μM GA were applied to the transgenic tobacco lines and the GUS activity was examined. The histochemical staining pattern revealed the effect of the GA treatments on the level of GUS activity in transformed tobacco leaves (Fig. 6b). As expected, the *GUS* gene driven by the *35SCaMV* promoter (positive control) was constitutively expressed in the transformed tobacco leaves under control and GA-treated conditions in comparison with negative control (pBI101 construct-lacking *35SCaMV* promoter) transgenic lines (Fig. 6b and c). However, low level of GUS activity driven by the *VvMIR160c* and *VvARF10* promoters 1 and 2 were observed in untreated (0 μM GA) transformed tobacco leaves (Fig. 6b and c). GUS activity increased markedly in *VvMIR160c* and *VvARF10* promoter 1-containing constructs (pBI121-*p1VvmiR160c-GUS* and pBI121-*p1VvARF10-GUS*) in the transgenic tobacco leaves-treated with 30 and 50 μM GA, especially at higher dose (50 μM GA) in comparison with the untreated transgenic tobacco leaves (Fig. 6b and c). However, GUS activity almost did not changed in *VvMIR160c* and *VvARF10* promoter 2-containing constructs (pBI121-*p2VvmiR160c-GUS* and pBI121-*p2VvARF10-GUS*) in transgenic tobacco leaves treated with 30 and 50 μM GA (Fig. 6b and c). Our data clearly confirmed that *VvMIR160c* and *VvARF10* promoter 1 region with 1500 bp contained GA-related cis-responsive elements, which is essential for inducing GA response.

### Verification of VvmiR160-mediated *VvARFs* cleavage roles by RLM-RACE and PPM-RACE

**Fig. 7:**
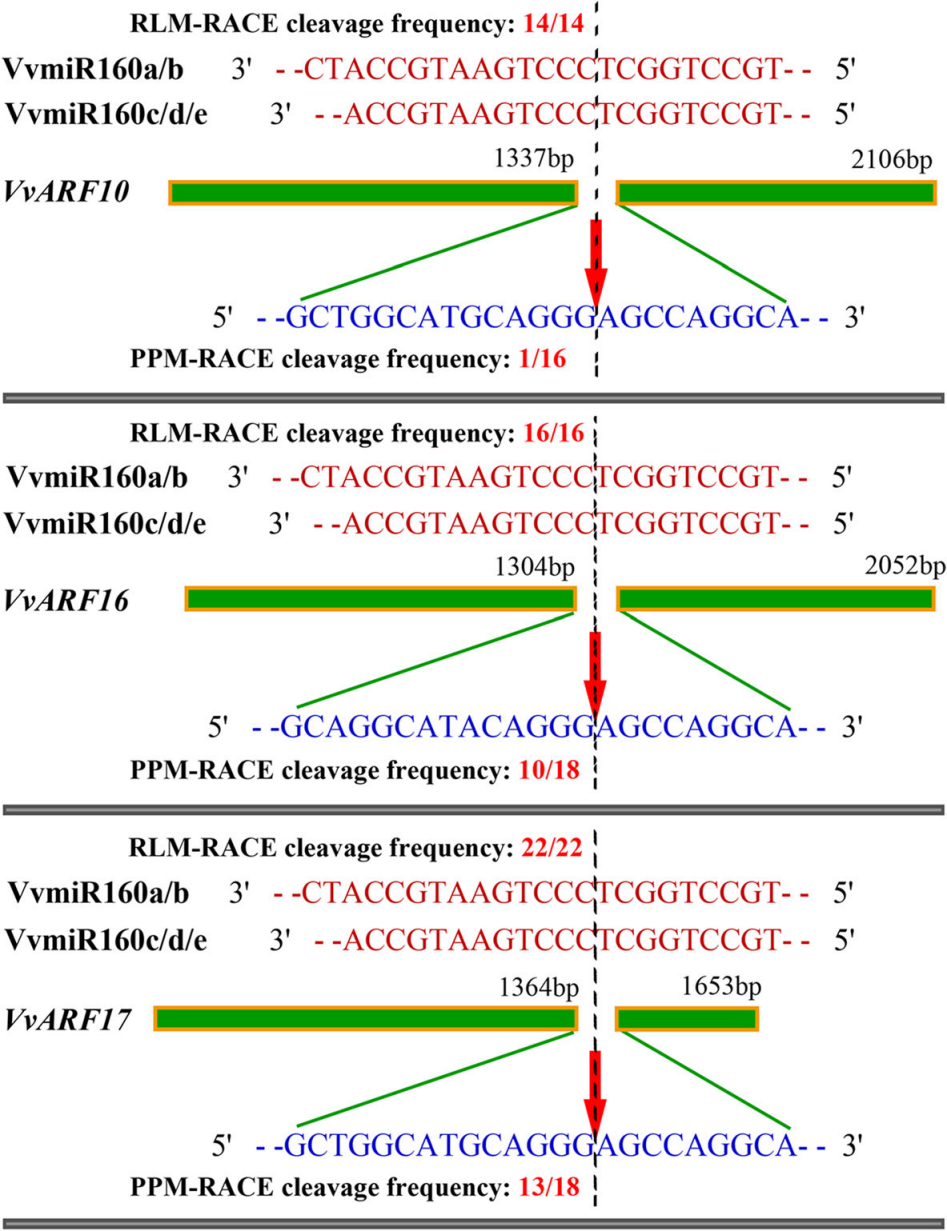
Mapping of the VvmiR160s-mediated cleavage sites on *VvARFs* by PPM-RACE and RLM-RACE. The red arrows indicate the cleavage sites of VvmiR160s on *VvARFs* identified by 5′ and 3′-ends of mRNA fragments cloned by PPM-RACE and RLM-RACE, respectively. The red and blue sequences represent the mature sequences of VvmiR160s and their target region sequences in *VvARFs*, respectively. Green strip denotes the remaining sequence outside the targeted region

The interaction between VvmiR160 family members and *VvARF* target genes, and their regulatory mechanisms in grape floral tissues were investigated by our modified RLM-RACE and developed PPM-RACE procedures [30]. First, from RLM-RACE, the sequencing of the amplified 3′-end products revealed only one cleavage site in the middle of complementary regions of VvmiR160 family members and *VvARF10/16/17* (Fig. 7). All the cleavage sites were mapped in the 9th site from the 5′- end of VvmiR160a/b/c/d/e (Fig. 7), indicating the specificity of cleavage sites of miRNAs, which is consistent with previous studies [30, 33, 34]. Next, our developed PPM-RACE was employed to further confirm the target genes of VvmiR160s and their cleavage sites. The sequencing of the amplified 5′-end products identified the same cleavage sites as those of the 3′-end sequencing in the RLM-RACE experiment. In addition, the cleavage frequencies of 5′-end product were less than those of 3′-end product, which might be attributed to the more stability of 3′-end fragments than 5′-end one [30, 33, 34]. The consistent results of both the RLM-RACE and PPM-RACE experiments confirmed that *VvARF10*, *VvARF16,* and *VvARF17* are the downstream target genes of VvmiR160a/b/c/d/e, and verified their cleavage interaction mode in grapevine inflorescence (Fig. 7). Although the cleavage sites of VvmiR160s were located on the same 9th position from their 5′-ends, these cleavage sites mapped on different positions in the respective *VvARF* target genes (Fig. 7). For instance, all cleavage sites of VvmiR160s were located at the 9th position from 5′-end of VvmiR160s, but located at the 1337th with 14/14 from the 5′-end for *VvARF10*, 1304th with 16/16 from the 5′-end for *VvARF16* and the 1364th with 22/22 from the 5′-end for *VvARF17* (Fig. 7).

### Differential expression profiles and correlation analysis of VvmiR160s and *VvARFs* during grape development

**Fig. 8:**
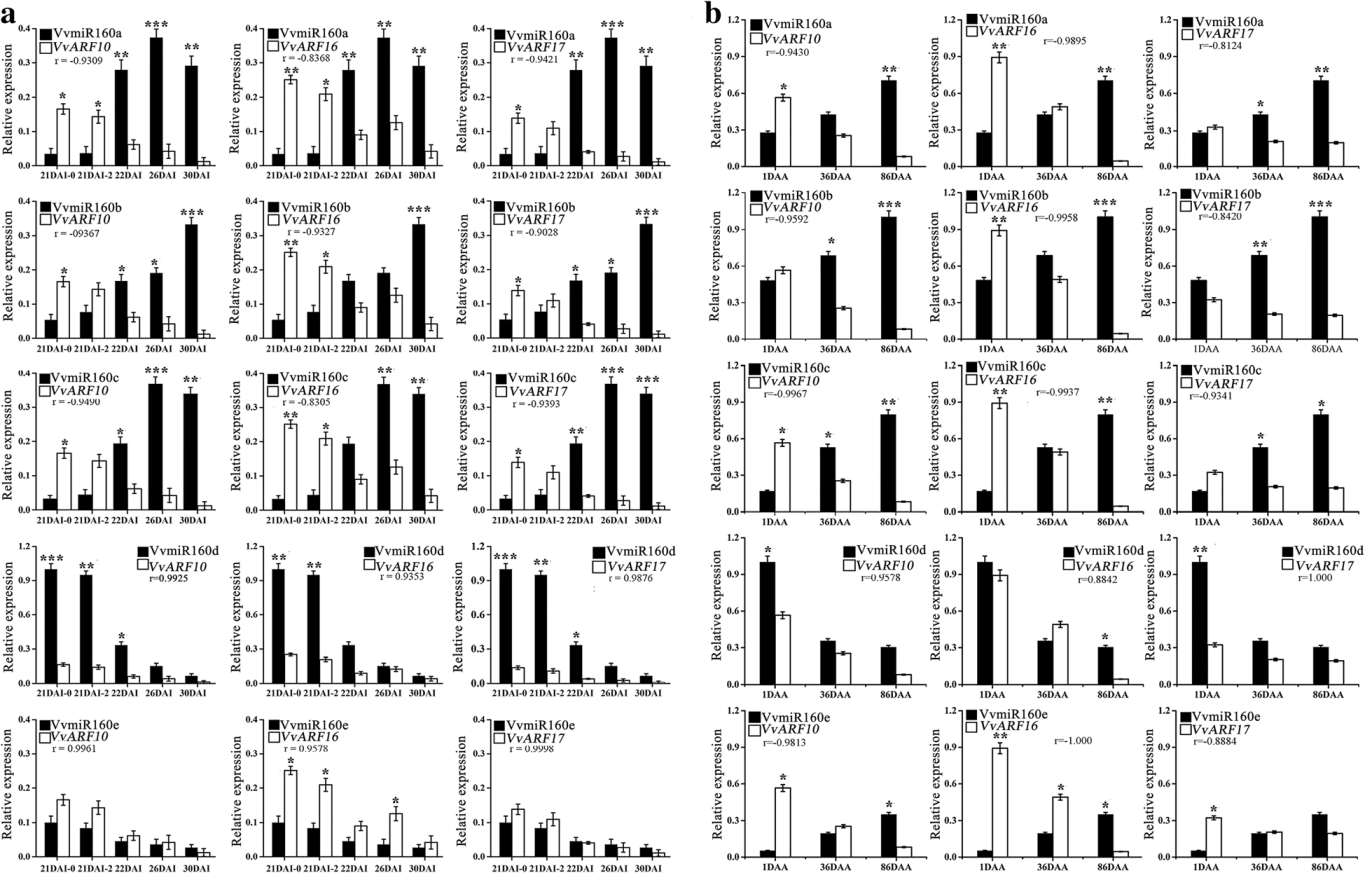
Characterization of *VvmiR160*:*VvARF* expression and Pearson correlation analysis during grape flower and berry development. **a** Characterization of VvmiR160:*VvARF* expression and Pearson correlation analysis during grape flower development. **b** Characterization of VvmiR160:*VvARF* expression and Pearson correlation analysis during grape berry development. The relative expression of VvmiR160a/b/c/d/e and the three *VvARF10/16/17* target genes at different stages [21 days after inflorescences (21DAI), 22DAI, 26DAI and 30DAI; 1 day after anthesis (1DAA), 36DAA, 86DAA] of floral and berry development. Pearson correlation coefficient (r) between *VvmiR160* and *VvARF* expression are indicated. Each experiment was repeated three times. ANOVA test was used to identify significant differences, Asterisks indicated statistically significant differences at (*P < 0.05; **P < 0.01; ***P < 0.001) as determined by Student’s t-test

The effect of the interaction between *VvmiR160s* and *VvARFs* during grape flower development was examined by measuring the spatio-temporal expression profiles of *VvmiR160s* and *VvARFs* under control condition (Fig. 8a). As shown in Fig. 8a, the expression level of VvmiR160 family could be classified into three groups. In group one, *VvmiR160a*/*b/c* exhibited the highest expression level during the late stage of floral development [from 22 days after inflorescence (22DAI) to 30DAI], and low expression during the early stage of floral development (Fig. 8a). In addition, *VvmiR160a* and *VvmiR160c* exhibited similar expression trend with the highest expression level has been detected at 26DAI, while *VvmiR160b* possessed the highest level at 30DAI (Fig. 8a). In group two, *VvmiR160d* exhibited a reverse expression trend relative to *VvmiR160a/b/*c, with high expression level has been detected at early floral development, followed by a gradual decrease till the late phase of floral development (Fig. 8a). In group three, *VvmiR160e* showed a low expression level at all stages, suggesting that *VvmiR160e* might have a slight role during grape floral development (Fig. 8a). Subsequently, the expression profiles of *VvmiR160a/b/c* exhibited one upward trend along with berry development [from 1 day after anthesis (1DAA) to 86DAA], and reached the high peak till maturation (86DAA); while *VvmiR160d* exhibited a reverse expression trend relative to *VvmiR160a/b/*c, and *VvmiR160e* showed a low expression level at all stages, suggesting that *VvmiR160e* might also have a slight role during grape berry development (Fig. 8b).

All three *VvARF10/16/17* target genes exhibited a reverse expression trend in comparison with *VvmiR160a/b/c*, where their expression levels increased during the early stage of flower/berry development and decreased gradually till the later stage (Fig. 8a and b). However, *VvARF10/16/17* expression profiles were similar to *VvmiR160d* expression during the different stages of grape flower/berry development (Fig. 8a and b). The Pearson correlation coefficient showed that *VvARF10*/*16*/*17* expression exhibited a highly negative correlations (*r* = − 0.83 to − 0.94 for flowers; *r* = − 0.81 to − 1 for berries) with *VvmiR160a/b/c* and positive correlation (*r* = 0.93 to 0.99 for flowers; *r* = 0.8842 to 1 for berries) with *VvmiR160d* (Fig. 8a and b). In general, the spatio-temporal expression and correlation analyses indicated that VvmiR160a/b/c were the key regulatory factors in grape floral and berry development through the negative regulation of *VvARF* expression.

### The expression profiles of VvmiR160s and *VvARFs* in response to GA treatment during grape development

**Fig. 9:**
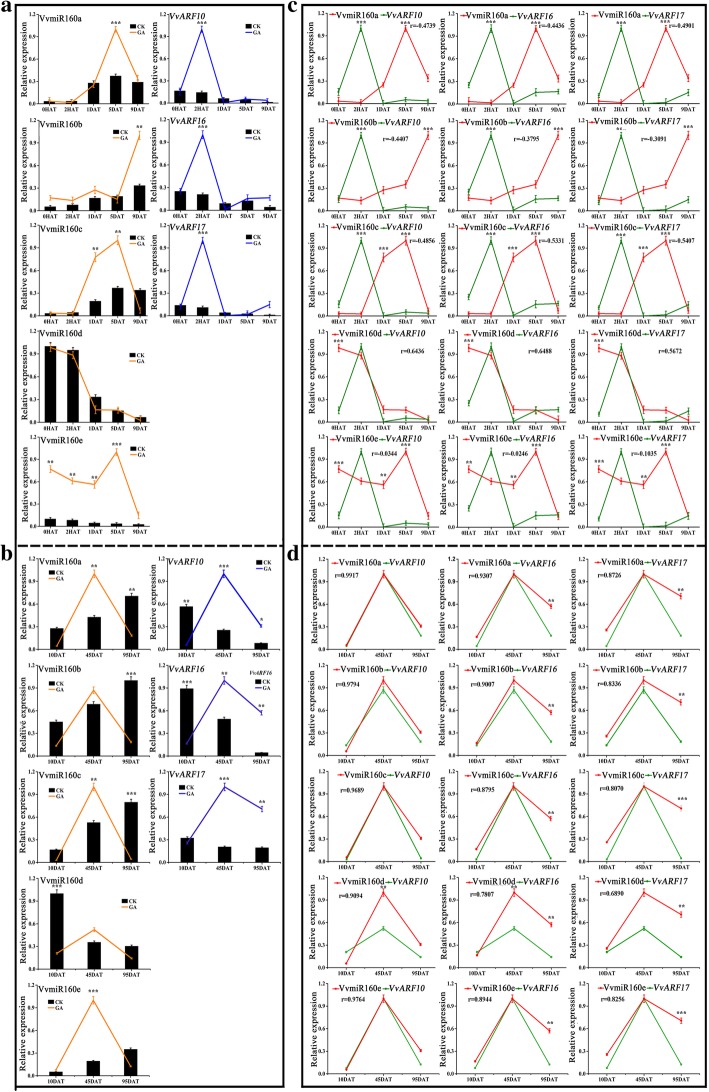
Differential expression of *VvmiR160* and *VvARF* target genes in response to GA application at different stages of floral and berry development. **a** Differential expression of *VvmiR160s* and their *VvARF* target genes in response to gibberellin (GA) treatment at different time points [0 h after treatment (0HAT), 2HAT, 1 day after treatment (1DAT), 5DAT, 9DAT] of floral development. **b** Differential expression of *VvmiR160s* and their *VvARF* target genes in response to gibberellin (GA) treatment at different time points (10DAT, 45DAT, 95DAT) of berry development. **c** Differential expression and Pearson correlation analysis of each member of VvmiR160 family and its respective *VvARF* target genes in response to GA treatment during floral development. **d** Differential expression and Pearson correlation analysis of each member of VvmiR160 family and its respective *VvARF* target genes in response to GA treatment during berry development. Each experiment was repeated three times. ANOVA test was used to identify significant differences, Asterisks indicate significant difference between GA-treated plants and respective untreated control (CK) plants at each time point as determined by Student’s *t*-test (*P < 0.05; **P < 0.01; ***P < 0.001)

GA is an essential factor for plant growth and development and the exogenous application of GA induced the expression level of *VvmiR160s* in plants [19, 29]. However, the relative effect of GA-mediated grape flower and berry development through *miR160s* remains elusive. In the present study, a comparative expression level of *VvmiR160s* and *VvARF* target genes in GA-treated and untreated control (CK) plants during floral development was carried out (Fig. 9a). The exogenous application of GA significantly up-regulated *VvmiR160a/b/c/e* expression at 5DAT and 9DAT in comparison with untreated control plants (Fig. 9a). However, there was no significant effect of GA treatment on *VvmiR160d* expression (Fig. 9a). These results indicated that GA is a positive long-term regulator of VvmiR160a/b/c/e during grape floral development, and the various members of miR160 family possessed diverse modes of response to GA. On the other hand, GA treatment induced *VvARF10*/*16*/*17* expression mainly at 2HAT (Fig. 9a), implying that these target genes displayed a short-term response to GA treatment during floral development. Unlike flower development, GA treatment almost significantly up-regulated the expression of VvmiR160s and three *VvARFs* genes at 45DAT (45 days after treatment; berry expansion) (Fig. 9b). In addition, the three *VvARFs* were slightly up-regulated at their expression levels by GA at the maturation stage (Fig. 9b).

Further, we compared the expression levels of VvmiR160s and their *VvARF* target genes under GA treatments, we revealed that in grape flowers, VvmiR160a/c and *VvARF10/16/17* expression exhibited some negative correlations (*r* = − 0.44 to − 0.53) in response to the short- and long-term effect of GA treatment in comparison with untreated controls (Fig. 9c); however, in berries, their expression did not possess the negative correlation (Fig. 9d). The correlation analysis indicated that GA might strength the regulatory roles of VvmiR160a/c on *VvARF10/16/17* during floral development. However, VvmiR160b and its targets had negative correlations only in the short-term during floral development, while VvmiR160e and its targets possessed apparent negative correlations in the long-term. GA had no effect on VvmiR160d expression, compared with untreated control plants, and both VvmiR160d and *VvmiR10/16/17* exhibited some positive correlation (*r* = 0.56 to 0.64) (Fig. 9c). In grape berries, VvmiR160a/b/c/d and *VvARF10/16/17* expression exhibited some positive correlations (*r* = 0.69 to 0.99) in response to GA (Fig. 9d). These results suggested that VvmiR160a/b/c/d displayed various modes in responsive to GA during grape floral and berry development.

**Fig. 10:**
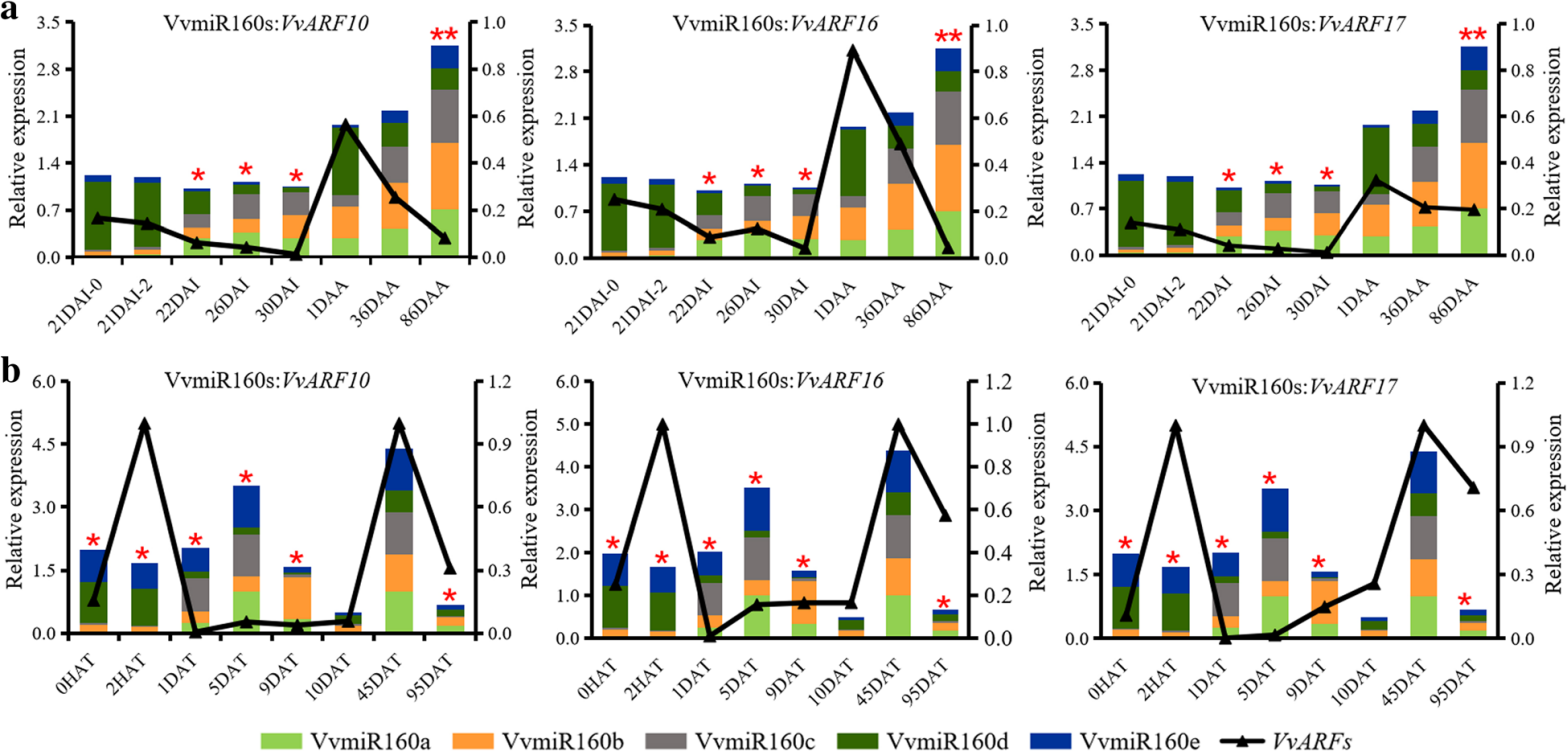
Pearson correlation analysis of total expression of *VvmiR160s* and their *VvARF* targets genes. **a** Correlation of total expression of *VvmiR160s* and their *VvARF* target genes during grape development [21 days after inflorescences (21DAI), 22DAI, 26DAI and 30DAI; 1 day after anthesis (1DAA), 36DAA, 86DAA]. **b** Correlation of total expression of *VvmiR160s* and their *VvARF* target gene s in response to gibberellin (GA) treatment at different time points [0 h after treatment (0HAT), 2HAT, 1 day after treatment (1DAT), 5DAT, 9DAT, 10DAT, 45DAT, 95DAT]. ANOVA test was used to identify significant differences, Asterisks indicated statistically significant differences at (*P < 0.05; **P < 0.01; ***P < 0.001) as determined by Student’s t-test

Considering the redundancy and complementary traits of miRNA’s function, the correlation analysis of the total expression levels of all members of VvmiR160 family and each of *VvARF* target genes was investigated (Fig. 10a and b). Results showed that the total expression of VvmiR160s had negative correlations with *VvARF10/16/17* target genes mainly at the late stages (22DAI, 26DAI, and 30 DAI, marked with an asterisk) of grape floral development under control (Fig. 10a), and the total expression of VvmiR160s had negative correlations with *VvARF10/16/17* target genes only at maturation (86DAA, marked with an asterisk) of grape berry development under control (Fig. 10a). While, under GA treatment, GA enhanced the negative regulatory effect of VvmiR160s on their target genes nearly at all stages (0HAT to 9DAT, marked with an asterisk) of grape floral development (Fig. 10b); however, no obvious effect of GA on the interaction of VvmiR160s and their target genes was observed during grape berry development (Fig. 10b). These results suggested that VvmiR160 family might possess some redundancy of functions, and indicated that GA is an essential factor for inducing the regulatory roles of VvmiR160s on their targets at short-term as well as long-term.

### Dynamic accumulation of cleavage products of *ARF10/16/17* during GA-induced grape floral development

**Fig. 11:**
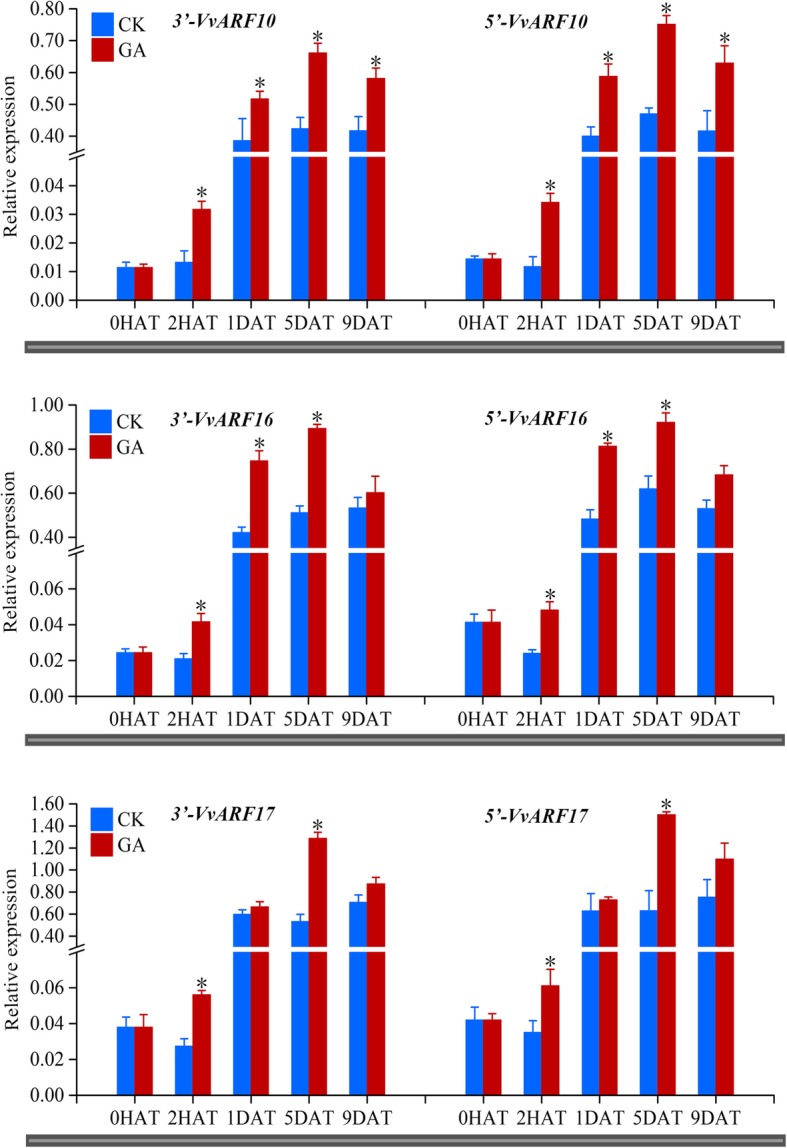
Accumulation patterns of 3′- and 5′-end cleavage products of miR160s cleaved *VvARF10/16/17* target genes in gibberellin (GA)-treated and untreated control (CK) plants at different stages of grape floral development. *VvARFs*-5’represents the accumulation of 5′-end cleavage products of *VvARFs* mediated by VvmiR160s using real-time qPCR; *VvARFs*-3′ represents 3′-end cleavage products. Each experiment was repeated three times. [0 h after treatment (0HAT), 2HAT, 1 day after treatment (1DAT), 5DAT, 9DAT]. ANOVA test was used to identify significant differences, Asterisks indicated statistically significant differences at (*P < 0.05; **P < 0.01) as determined by Student’s t-test

Detecting the accumulation of cleavage products of VvmiR160s and *VvARFs* during grape floral development could be helpful to determine the spatio-temporal variation of their cleavage roles. Our modified RLM-RACE and developed PPM-RACE protocols together with qRT-PCR were employed to monitor their 3′- and 5′-cleavage products under GA treatment. Results showed that GA treatment promoted greater accumulation of 3′- and 5′-cleavage products of *VvARF10*/*16*/*17* in comparison with untreated control plants (Fig. 11). GA induced the highest accumulation of 3′- and 5′-cleavage products of *VvARF10/16/17* at 5DAT. However, GA slightly induced 3′- and 5′-cleavage products at 2HAT, indicating that GA exhibited a profound long-term effect on VvmiR160s’ cleavage roles rather than a short-term one (Fig. 11). Although, the accumulation of 5′-cleavage products of *VvARF10/16/17* was almost similar to 3′-cleavage products at various phases in GA-treated and untreated control plants (Fig. 11), the 3′-cleavage products were slightly higher, which might be due to the high stability of 3′-cleavage products compared with 5′-cleavage products as previously reported [30, 33, 34].

## Discussion

The effects of exogenous GA application on the induction of flower development, seedless cultivars, berry enlargement and fruit ripening of grapevine have recently been reported [18, 20]. However, the molecular mechanisms by which GA-signaling mediates flower and fruit set initiation in grapevines are still not fully understood. In recent years, miRNAs have been identified as a critical regulatory factor of gene expression through indirect, or direct transcriptional and post-transcriptional gene silencing to modulate the activity of the gene network underlying various developmental and stress-responsive programs [3, 9, 14, 21]. Several miRNA families involved in the modulation of barley (*Hordeum vulgare*), *Arabidopsis*, cotton (*Gossypium hirsutum*) and tomato flower development have been identified and released in miRBase [10, 11, 24, 35–37]. For example, a recent study has reported that targeting of *ARF10/16/17* by miR160 is indispensable for various aspects of AUX-mediated floral organ abscission as well as ovary patterning in tomato plant [24]. Similarly, a loss-of-function of *Arabidopsis atmir160a* plants changed the expression of *ARF16/17* and displayed different phenotypes, including irregular flowers, reduced fertility, aberrant seeds and floral organs inside siliques [17]. Our previous studies [15, 29] revealed that the exogenous application of GA_3_ induced the expression of several miRNA families, including *miR160s* and *miR159s* during grape floral development, suggesting that these miRNAs might play important roles in grape floral development and parthenocarpic process. In the present study, we utilized the grapevine parthenocarpy system to investigate the regulatory functions of *VvmiR160s*-*VvARFs* in response to GA-mediated grapevine floral development. The exogenous application of GA_3_ induced a variety of intriguing floral and berry morphology in ‘Rosario Bianco’ cultivar, including dark-yellow anthers, long filaments, short styluses, big ovaries and high seedless ratio, long berry spikes and grains longitudinal diameter relative to untreated control plants (Fig. 1a and b). Our result was in line with the recent study by Wang et al. [15] which demonstrated that GA_3_ application induced distinct floral morphology in grapevine cv. ‘Zuijinxiang’, confirming that GA-signaling is a crucial factor for the positive regulation of grape parthenocarpy and fruit set development.

Based on our previous high-throughput sequencing of GA-induced grapevine parthenocarpy [15, 29], the precise sequences of *VvmiR160a/b/c/d/e* and their *VvARF10/16/17* target genes were isolated, sequenced and mapped into the corresponding chromosomes (Figs. 2 and 3). Although miRNAs are evolutionarily conserved among different plant species [38], our *VvmiR160a/b* exhibited two nucleotide shortage in comparison with homologous sequences from wine grapevine cv. ‘Pinot Noir’ in miRBase 21.0, while those of *VvmiR160c/d/e* were consistent in both grapevine cultivars (Fig. 2b). Our previous study revealed that about 85.2% of the known miRNAs had single cleavage sites on their target genes, whereas the remaining 14.8% of the known miRNAs had two to three cleavage sites, and these cleavage sites were mainly mapped on the 9th, 10th, or 11th nucleotide position from the 5′-ends of the corresponding miRNAs [39]. According to the base-pair complementary degree between *VvmiR160s* and *VvARF*s using 5′-RLM-RACE and 3′-PPM-RACE technology [30], we demonstrated that all *VvmiR160a/b/c/d/e* had the same cleavage site on 9th position from the 5′-end (Fig. 7), whereas their corresponding cleave sites on *VvARF10/16/17* were variables and located at the 1337th with 14/14, 1304th with 16/16 and 1364th with 22/22 from the 5′-end for *VvARF10*, *VvARF16* and *VvARF17*, respectively (Fig. 7). These results suggested that the cleavage sites possessed the specific trait of miRNA sites’ recognition, similar to previous reports that miRNAs cleaved their targets at specific sites from their 5′-ends like 9th, 10th and 11th [30, 33, 34]. PPM-RACE results further showed higher accumulation levels of 3′-end cleavage products than those of RLM-RACE 5′-end cleavage products. The accumulation of 3′-end cleavage products could be attributed to the rapid degradation of 5′ -RNA fragment generated after miRNA-directed cleavage, while the 3′ -RNA fragments are more stable, similar to previous reports in apple, orange, and peach [30, 38].

### Conserved regulatory roles of miR160 on their *ARF* target genes across diverse plant species

The phylogenetic tree of our VvARFs and orthologues ones demonstrated an orthologous relationship between VvARFs and AtARFs, SlARFs and JrARFs (Fig. 4a), suggesting that these ARF proteins might have descended from a common ancestor and can have conserved functions. The amino acid sequences of the three VvARF10/16/17 contained a conserved N-terminal B3 and AUX_resp DBD, and C-terminal AUX/IAA domain (CTD: domain III/IV), whereas VvARF17 and other orthologues ARF17 members showed the absence of C-terminal Aux/IAA domains (Fig. 4c). B3-type and AUX_resp DBD are essential for the interaction between ARFs and TGTCTC AuxREs, whereas AUX/IAA domain facilitates protein-protein interactions via homo- and hetero-dimerization among the members of both the ARF and Aux/IAA families [40–42]. The Auxin_resp domain is an evolutionary step in plants, and freshwater algae ancestors of land plants are reported to contain precursor ARF-like proteins with III/IV and B3 domains but lack the Auxin_resp domain [22]. In addition, gene structure analysis indicated that the coding sequences of all the *VvARF* genes are disrupted by introns; however, the total number of introns was varied among different *VvARF* members (Fig. 4b). The exon-intron structures of most homologous *ARF* genes had a similar distribution of exon-intron structures, which provide vital evidence to support the reliability of the phylogenetic analysis of *VvARFs* and their homologous *ARFs* in *Arabidopsis*, tomato and other species (Fig. 4a and b) and suggest a potential conserved function.

Furthermore, promoter motifs related to light, hormone, tissue-specific, circadian and stress-responsive *cis*-elements of VvmiR160s and their *VvARF* targeted genes exhibited high similarity, suggesting that their function is similar to a certain extent (Fig. 5a). Likewise, the promoter analysis of miR160s isolated from *Dimocarpus longan* showed that *cis*-elements responsive to stimuli such as light, hormone and circadian were also detected [32, 43]. In the present work, all the promoter regions of VvmiR160a/c/d/e (except for VvmiR160b) and *VvARFs* contained *cis*-elements related to various hormonal response (GA, IAA, SA, ABA, MeJA and ET), indicating that VvmiR160s are associated with hormone transduction during grape development. Among these hormone-responsive motifs, GA-responsive *cis*-element was highly abundant in *VvmiR160s* and *VvARFs,* in addition, the transient expression experiments and GUS activity assays of *VvmiR160c* and *VvARF10* promoters showed they were probably positively regulated by GA, which added further evidence regarding the essential roles of *VvmiR160*-*VvARF* pairs in grape development through GA-signaling. Likewise, number of copies of GA-responsive elements were also detected in the promoter regions of *Glycine max* (*GmmiR160f/g/h*) and *Manihot esculenta* (*MemiR160a*) and their *GmARF6/8/17/18* and *MeARF10* targeted genes, respectively [29, 44]. In general, these results clearly demonstrated the significant role of VvmiR160s in the regulation of GA-signaling through *VvARFs*.

### Characterization of the expression patterns responsive to GA of various VvmiR160s:*VvARF* pairs during grape development

Multiple members of miRNA family and their target genes can form miRNA-mediated regulatory networks with some redundancy and complementarity functions, and different miRNA family members had diverse spatio-temporal expression modes [45]. Changes in the expression profiles of *VvmiR160*:*VvARF* pairs during grape development were presented in Fig. 8. Recent studies have shown that miR160s negatively regulate *ARF10/16/17* in *Arabidopsis*, tomato, longan and populous [46–49]. However, the regulatory roles of miR160s in grape floral and berry development are unknown. In the present study, the spatio-temporal expression of different members of *VvmiR160*s showed that *VvmiR160a/b/c* exhibited a significant increase in their expression levels during GA-mediated floral development (Fig. 9a). However, *VvARF10/16/17* targeted genes displayed a reverse expression trend compared with *miR160a/b/c*. Our result was in accordance with Lin et al. [32] demonstrated that GA treatment induced longan *Dlo-miR160a/d* expression and down-regulated *DlARF10/16/17* during the middle and late stage of somatic embryogenesis. Similarly, *foc* mutant *Arabidopsis* seedling with a *Ds* transposon insertion in the 3′ regulatory region of *atmir160a* exhibited down-regulation in *atmir160a* expression and up-regulation in *AtARF10/16/17* in response to IAA treatment, and *foc Arabidopsis* mutant showed irregular flower phenotyping and reduced fertility [17]. Likewise, the knockdown of tomato *SlmiR160* expression using a short tandem target mimic (*STTM160*) displayed abnormal floral organ abscission, which was associated with the up-regulation of *SlARF10A/B* and *SlARF17* [24]. Our results and the above reports, clearly demonstrated that VvmiR160s are crucial for GA-induced grapevine floral development through the negative regulation of *VvARF10/16/17* expression. In grape berries, the expression profiles of VvmiR160a/b/c exhibited one upward trend along with berry development, and reached the high peak till maturation (Fig. 8b), likewise, the expression patterns of miR160a was gradually increased as fruit develops and ripens in blueberry [50]. In addition, our spatio-temporal expression of GA-induced grapevine shown that *VvmiR160d* expression was up-regulated at the early stage but down-regulated at the middle and later stage of GA-induced grape floral development, suggesting that VvmiR160d is acting as a repressor for grape floral development (Fig. 9a). Also, *VvmiR160e* expression displayed low expression level at all stages, implying that VvmiR160e has a minor role in grapevine floral and berry transition (Fig. 9a and b). We further carried out a comparative expression profiles of *VvmiR160s* and *VvARF* targeted genes between GA-treated and untreated control (CK) grapevine to validate the significant role of GA treatment on grapevine floral/berry development through the VvmiR160-*VvARF* regulatory network (Fig. 9a and b). The expression trend of VvmiR160s and *VvARFs* at different stages under GA-treated and untreated grapevine plants was quite similar during grape floral development, however, GA treatment induced the expression of these respective genes much higher than their expression level in the untreated control plants (Fig. 9a). In contrast, GA treatment almost significantly up-regulated the expression of VvmiR160s and three *VvARFs* genes at berry expansion (Fig. 9b). It worth noting that IAA stimulates GA biosynthesis in pea pericarp [51, 52] through a process mediated by one or more signaling elements, and that GA metabolism interacts with IAA signaling independent of feedback regulation [53], when the AUX signaling pathway was blocked. These results could provide significant information for further gaining the functions of VvmiR160s and their target *VvARFs* during GA-induced grape parthenocarpy process.

## Conclusion

This study examined the morphological changes of the ‘Rosario Bianco’ floral and berry development during grapevine parthenocarpy process-induced by the exogenous GA_3_ application. The precise sequences of VvmiR160s in ‘Rosario Bianco’ grape flowers and their *VvARF10/16/17* target genes were predicted, cloned and verified. Furthermore, the conserved and diversification phylogenetic relationships of VvARF with other orthologues from various plant species were carried out. All VvmiR160s (except VvmiR160b) and *VvARF10/16/17* had showed the common cis-elements responsive to GA, and this was confirmed during our experiments, which support their function in GA-mediated grape parthenocarpy. The spatio-temporal expression and correlation analysis indicated that VvmiR160a/b/c are the key factors that negatively regulate *VvARF10/16/17* target genes during GA-induced grapevine floral and berry development. The negative regulation of *VvARF10/16/17* expression by *VvmiR160a/b/c* as key regulatory factors was critical for GA-mediated grape parthenocarpy, and provided a significant step forward in understanding the molecular mechanisms of VvmiR160s and their *VvARFs* for molecular breeding of high-quality seedless berry. Further studies are required to identify the specific downstream genes that are targeted by the corresponding *ARFs* in each process.

## Methods

### Plant materials and GA_3_ treatment

Basing on the preliminary study and the necessity of performing the related research work, 6-year-old trees of grapevine cv ‘Rosario Bianco’ (*Vitis vinifera*), an elite grape cultivar widely cultivated in China, were used in this study. The plant materials were grown under common field conditions at the Jiangsu Vocational College of Agriculture and Forestry grape farm, Jurong, China (our partners, we have a long-term relationship). Referencing to the production experience and variety characteristics, the grape clusters were soaked in 50 mg L^− 1^ GA_3_ (dissolved in 0.2% Tween-20) for 30s, and water-treated grape clusterswere used as a control at 21 days after inflorescences. The GA-treated plants reached anthesis at 9 days after treatments. Inflorescence and berry samples were randomly collected from different branches of different treatment groups at different time points [0 h, 2 h, 1 , 5 , 9 , 10 , 45 and 95 days after treatment (0HAT, 2HAT, 1DAT, 5DAT, 9DAT, 10DAT, 45DAT and 95DAT respectively). Samples were used for phenotypic characters, and the other part of samples were immediately frozen in liquid nitrogen and stored at − 80 °C until use.

### RNA extraction, high-molecular-weight RNA, low-molecular-weight RNA isolation and cDNA synthesis

Total RNA was isolated from 200 mg grapevine tissues mentioned above using our modified CTAB method [30]. Low-molecular-weight RNA (LMW RNA) and high-molecular-weight RNA (HMW RNA) were separated with 4 M LiCl. The mixtures of diverse stage LMW RNA samples were loaded into Poly(A) tails using Poly(A) Tailing kit from TAKARA. The Poly(A)-tailed LMW RNAs were further ligated to 5′ adapter (5′-CGACUGGAGCACGAGGACACUGACAUGGACUGAAGGAGUAGAAA-3′) using T4 RNA ligase (Invitrogen, Carlsbad, CA). Then, these LMW RNAs with Poly (A) and 5′-adapter were reversetranscripted into cDNAs for miR-RACE clone. Mixtures of diverse stage HMW RNA samples were directly reverse transcripted into cDNAs for qRT-PCR. All cDNAs were stored at − 80 °C before use.

### The clone of the precise sequences of VvmiR160s in the floral tissues of grape cv. ‘Rosario Bianco’ by miR-RACE

The mixtures of diverse stage LMW RNA samples were loaded into Poly(A) tails using Poly(A) Tailing kit from TAKARA. The Poly(A)-tailed LMW RNAs were further ligated to 5′ adapter (5′-CGACUGGAGCACGAGGACACUGACAUGGACUGAAGGAGUAGAAA-3′) using T4 RNA ligase (Invitrogen, Carlsbad, CA). Then, these LMW RNAs with Poly (A) and 5′-adapter were reverse transcripted into cDNAs for clones of VvmiR160 sequences by miR-RACE [30].

### Prediction of the targets for VvmiR160s, identification of the degree of complementarity and related parameters of VvmiR160s:*VvARF* pairs

Based on the precise sequences of five VvmiR160 members, we employed Multiple Align (BioXM software) to align these sequences, revealing two unique mature sequences (VvmiR160a/b and VvmiR160c/d/e). The miRNA unique sequences were further used as the benchmark to blast search in grape transcript database V2 (http://genomes.cribi.unipd.it/grape/) for prediction of target genes for VvmiR160s. Furthermore, the identification of the degree of complementarity, action modes, and other related parameters were characterized using the psRNATarget software (http://plantgrn.noble.org/psRNATarget/).

### Mapping of mRNA cleavage sites using RLM-RACE and PPM-RACE

To map miRNA-mediated cleavage products, our earlier modified RLM-RACE and developed PPM-RACE were employed to determine their 3′- and 5′-end cleavage products. Based on our previous report [30], HMW RNAs have been added to the Poly(A) (Ambion, Austin, TX) and ligated to 5′ adapter (5′-CGACUGGAGCACGAGGACACUGACAUGGACUGAAGGAGUAGAAA-3′) using T4 RNA ligase (Invitrogen, Carlsbad, CA), respectively. Then Poly (A)-tailed HMW RNA and adapter-ligated HMW RNA were recovered by phenol/chloroform extraction followed by ethanol precipitation. Poly (A)-tailed HMW RNA and adapter-ligated HMW RNA were further reverse transcripted into cDNAs for PPM-RACE and RLM-RACE. The amplification products were gel purified, cloned, and sequenced, and at least eight independent clones were sequenced.

### Distribution of VvmiR160s and target *VvARF* genes in the precursors/chromosomes

The precursors of VvmiR160s were downloaded from the plant miRBase 21.0 (http://www.mirbase.org/). Mfold online tool (http://unafold.rna.albany.edu/) were applied to fold their hairpin structures. All five VvmiR160s and three *VvARF* genes were mapped to grapevine chromosomes based on information available at the Grape Genome CRIBI website (http://genomes.cribi.unipd.it/grape/). The map was drafted using MapInspect software (http://www.plantbreeding.wur.nl/uk/software- mapinspect.html).

### Phylogenetic analysis

The software MEGA version 7.0.21 and ClustalX2 were employed to conduct the phylogenetic analysis. Homologs of *VvARF10, VvARF16, VvARF17* were identified by a blast search in the NCBI databases (https://blast.ncbi.nlm.nih.gov/Blast.cgi) using amino acid sequences of VvARF10/16/17. Multiple alignmentswere performed using ClustalX2, and the unrooted phylogenetic trees were constructed with MEGA 7.0.21 software using the Neighbor-Joining method, and the bootstrap test was carried out with 1000 replications.

### Analysis and distribution of exon/intron structures of *ARFs* in nine species

The exon/intron organization of *VvARF* genes was determined by comparing predicted coding sequences with their corresponding genomic sequences using the GSDS software (http://gsds.cbi.pku.edu.cn).

### Protein domains and their physicochemical properties analysis of target *ARFs* in various species

The Domains & Structures website (https://www.ncbi.nlm.nih.gov/guide/domains- structures/) were used to analyze protein domains of target genes, and their homologs sequences. Then, the IBS (Illustrator for Biological Sequences) software version 1.0.3 was used to draw the orthodox domains of ARF proteins. Besides, all target ARF protein sequences were considered and used in further analysis. Number of amino acids, isoelectric point, aliphatic index, grand average of hydropathicity, the molecular weight of deduced polypeptides were calculated by using tools provided at the ExPasy website (http://web.expasy.org/protparam/).

### Motif composition analysis of ARF proteins in nine species

The identification of motifs in the ARF protein sequences was performed with the MEME 5.05 online program (http://meme-suite.org/tools/meme). The optimized parameters of MEME were employed as follows: number of repetitions, any; maximum number of motifs, 20; and the optimum width of each motif, between 6 and 50 residues.

### Cis-element analysis of the promoters from *VvMIR160s* and target *VvARFs*

From the grape genoscope database (http://www.genoscope.cns.fr/externe/GenomeBrowser/Vitis/), we obtained the promoters (approximately 1500 bp upstream of genes) of *VvMIR160* (VvmiR160’s precursors) and *VvARFs*, and employ the Plantcare software (http://bioinformatics.psb.ugent.be/webtools/plantcare/html/) to predict the motif elements in these promoters.

### Construction of the expression vector and *Agrobacterium*-mediated tobacco transient transformation

The promoter sequences of *VvMIR160c* and *VvARF10* were isolated. The primers used were described in Additional file 6: Table S4. To develop pBI121-*p1VvMIR160c-GUS* and pBI121-*p1VvARF10-GUS* constructs, 1.5 kb promoter regions of *VvMIR160c* and *VvARF10* were independently cloned and fused with *GUS* reporter gene to replace the *35SCaMV* promoter in pBI121 binary vector. With respect to pBI121-*p2VvMIR160c -GUS* construct, 1442 bp promoter 2 fragment region of *VvMIR160c* without including the GA response element (59–1500 bp) was cloned into pBI121 to replace the *35SCaMV* promoter and was used to drive the *GUS* reporter gene. Similarly, for pBI121-*p2VvARF10-GUS* construct, 937 bp promoter 2 fragment region of VvmiR160c without GA response element (564–1500 bp) was cloned into pBI121 to replace the *35SCaMV* promoter and was used to drive the GUS reporter gene.

A single colony of *Agrobacterium tumefaciens* EHA105 (containing the recombinant plasmid) was cultured in YEB liquid medium supplied with rifampin (50 μg mL^− 1^) and kanamycin (50 μg mL^− 1^). After culturing, the bacteria were pelleted and resuspended in suspension buffer (10 mM MES, 10 mM MgCl2, pH 5.6). The bacterial.

suspension was then adjusted to OD600 = 0.5, and 100 μmol L^− 1^ acetosyringone was added before the suspension was used for infiltration, and left to stand at room temperature for 4 h. The leaves of 6-week-old tobacco plants were infiltrated with the bacterial suspension by injection. Tiny holes were made in the tobacco leaves using syringe needles. The bacterial suspension was then injected into those tiny holes with a needleless syringe. The infiltrated seedlings were then moved back to the environmental chamber and kept in the dark for 3 d. Treatment on tobacco leaves with 30 μM GA and 50 μM GA were performed 2 days later. The uniformly sized leaves were used in infiltration experiment, and the experiment was repeated three times.

### GUS assay

The infiltrated leaves were punched and then histochemical GUS staining of infiltrated leaves was performed as described by Jia et al. [54]. Leaves were then immersed in ethanol to remove Chl. Quantification of GUS activity was determined using the fluorometric 4-methylumbelliferyl-b-D-glucuronide (MUG) method. One unit of GUS activity was defined as 1 nM of 4-methylumbelliferon (4-MU) generated per minute per milligram of soluble protein. Three leaves were infiltrated for each construct in each independent experiment, and then combined to detect GUS activity.

### Expression analysis of VvmiR160s by qRT-PCR

The template for quantitative real-time polymerase chain reaction (qRT-PCR) was the cDNA for poly(A)-tailed small RNA mentioned above. To amplify the VvmiR160a/b/ c/d/e from the reverse-transcribed cDNAs, we used the precise sequences of VvmiR160 family members as the forward primer and R16328 (ATTCTAGAGGCCGAGGCGGCCGACATG) as the reverse primer [30]. qRT-PCR was performed with SYBR Premix Ex Tap™ kit (TaKaRa, Dalian, China), using Light Cycler®480 II (Roche, Switzerland). PCR cycling conditions consisted of an initial denaturation step at 95 °C for 30s, followed by 40 cycles at 60 °C for 20s and 95 °C for 5 s. The 5.8 s rRNA was used as a reference gene. Relative expression level was calculated with the formula 2^−ΔΔCT^ = normalized expression ratio. Each PCR assay was carried out by three biological replicates, and each replicate corresponded to three repeats of separate experiments. All primers were listed in Additional file 6: Table S4.

### Real-time PCR of the *VvARFs* expression

The expression of *VvARF10/16/17* was assayed by qRT-PCR as previously described [30]. The reverse transcription product was amplified using gene-specific primers that overlapped the known or predicted cleavage site. Reactions were performed in triplicates using the Light Cycler®480 II. The *Actin* gene was used as a reference gene in the qRT-PCR detection of mRNAs. Relative expression level was calculated with the formula 2^−ΔΔCT^ = normalized expression ratio. Each PCR assay was carried out in three biological replicates. The experiments were set the three repeats. All primers were listed in Additional file 6: Table S4.

### Statistics

ANOVA test was used to identify significant differences in floral morphology and gene expression between GA_3_-treated and untreated grape plants. Asterisks indicate a significant difference between GA-treated plants and respective untreated control (CK) plants at each time point as determined by Student’s *t*-test (*P < 0.05; **P < 0.01).

## Additional files

Additional file 1: **Figure S1.** Phylogenetic tree of three ARF domains based on an alignment of grapevine and *Arabidopsis*. The phylogenetic tree was generated with MEGA 7.0.21 software using the neighbor-joining method. Bootstrap values from 1000 replicates are indicated at each branch. (TIF 2006 kb)

Additional file 2: **Figure S2.** The open reading frame (ORF) and amino acid sequences of *AUXIN RESPONSIVE FACTOR (VvARF)10/16/17* in grapevine. Asterisk indicates the stop codon. (TIF 6656 kb)

Additional file 3: **Table S1.** Protein domains of ARFs and their physicochemical properties. Noaa, Number of amino acids; pI, isoelectric point; Ai, aliphatic index; GRAVY, grand average of hydropathicity; MW, molecular weight. (DOCX 21 kb)

Additional file 4: **Table S2.** All the motif information of *VvMIR160s* precursor genes and their targeted *VvARF* genes’ promoter.The Plantcare software (http://bioinformatics.psb.ugent.be/webtools/plantcare/html/) was used to predict the motif elements of these genes’ promoter. (DOCX 24 kb)

Additional file 5: **Table S3.** Motif elements related to hormone signals of MIR160s precursors’ genes and their targeted *ARFs* pairs in grapevine. The Plantcare software (http://bioinformatics.psb.ugent.be/webtools/plantcare/html/) was used to predict the motif elements of these genes’ promoter. (DOCX 16 kb)

Additional file 6: **Table S4.** List of the primers used for experiments. (DOCX 16 kb)

## Abbreviations

ABA: Abscisic acid
ARF: AUXIN RESPONSIVE FACTOR
AUX: Auxin
AuxREs: AUXIN RESPONSEELEMENTs
Chr: Chromosome
ET: Ethylene
*foc*: *floral organs in carpel*
GA: Gibberellin
HMW RNA: High-molecular-weight RNA
IAA: Indole acetic acid
LMW RNA: Low-molecular-weight RNA
MeJA: Methyl jasmonate
ORF: Open reading frame
PPM-RACE: Poly (A) polymerase -mediated 3′ rapid amplification of cDNA ends
RLM-RACE: RNA-ligase-mediated Rapid Amplification of cDNA Ends
SA: Salicylic Acid
SLR1: SLENDER RICE 1
STTM: Short tandem target mimic
