# Supplementary figures and images for: VvmiR160s/VvARFs interaction and their spatio-temporal expression/cleavage products during GA-induced grape parthenocarpy
Source: BMC Plant Biol. 2019 Mar 21;19:111. doi: 10.1186/s12870-019-1719-9 (PMC6429806; doi:10.1186/s12870-019-1719-9)

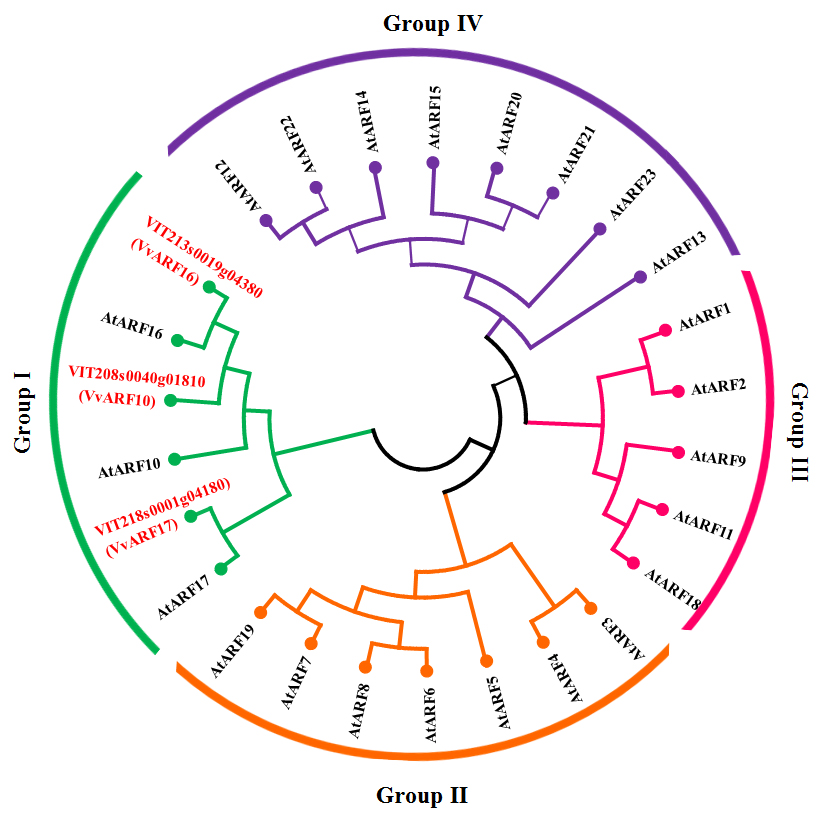

Supplement: Supplementary file 1 — Figure S1. Phylogenetic tree of three ARF domains based on an alignment of grapevine and Arabidopsis. The phylogenetic tree was generated with MEGA 7.0.21 software using the neighbor-joining method. Bootstrap values from 1000 replicates are indicated at each branch. (TIF 2006 kb) [file 12870_2019_1719_MOESM1_ESM.tif]

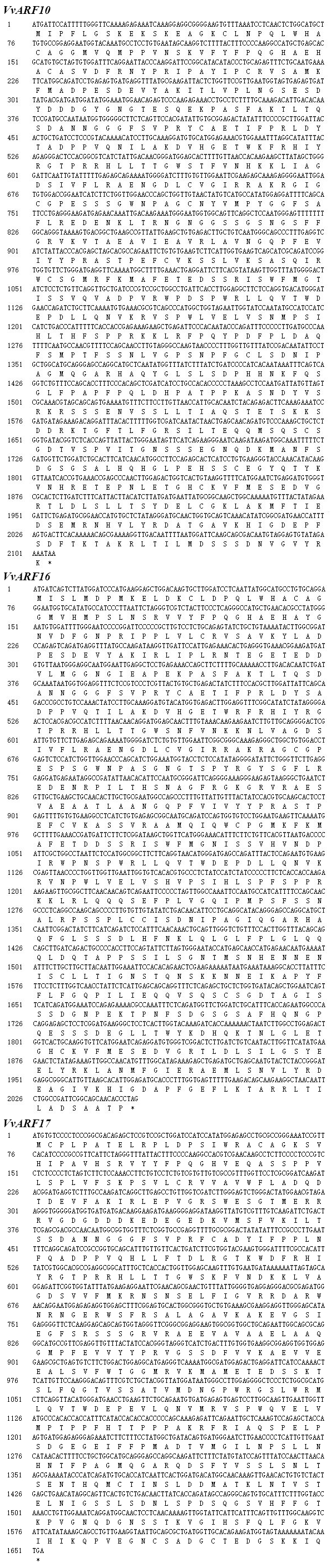

Supplement: Supplementary file 2 — Figure S2. The open reading frame (ORF) and amino acid sequences of AUXIN RESPONSIVE FACTOR (VvARF)10/16/17 in grapevine. Asterisk indicates the stop codon. (TIF 6656 kb) [file 12870_2019_1719_MOESM2_ESM.tif]
