# Supplementary material for: VvmiR160s/VvARFs interaction and their spatio-temporal expression/cleavage products during GA-induced grape parthenocarpy
Source: BMC Plant Biol. 2019 Mar 21;19:111. doi: 10.1186/s12870-019-1719-9 (PMC6429806; doi:10.1186/s12870-019-1719-9)
Supplement: Supplementary file 3 — Table S1. Protein domains of ARFs and their physicochemical properties. Noaa, Number of amino acids; pI, isoelectric point; Ai, aliphatic index; GRAVY, grand average of hydropathicity; MW, molecular weight. (DOCX 21 kb) [file 12870_2019_1719_MOESM3_ESM.docx]

**Additional file 3: Table S1**. **Protein domains of ARFs and their physicochemical properties.**

| Classification | | Proposed Gene Name | Annotation ID | Noaa | MW (KDa) | pI | Ai | GRAVY | *ARF* domain | |
| --- | --- | --- | --- | --- | --- | --- | --- | --- | --- | --- |
|  |  |  |  |  |  |  |  |  | Domain name | Interval |
| Group I | Subgroup I | PpARF10 | XP_020421973.1 | 729 | 80545.2 | 6.09 | 66.76 | -0.403 | B3 | 149-250 |
|  |  |  |  |  |  |  |  |  | Auxin_resp | 318-401 |
|  |  | PpARF16 | XP_007208053.1 | 703 | 77227.32 | 6.25 | 65.62 | -0.424 | B3 | 121-222 |
|  |  |  |  |  |  |  |  |  | Auxin_resp | 290-373 |
|  |  | PaARF10 | XP_021817874.1 | 699 | 76985.17 | 6.25 | 66.27 | -0.422 | B3 | 121-222 |
|  |  |  |  |  |  |  |  |  | Auxin_resp | 288-371 |
|  |  | PmARF10 | XP_008243945.1 | 701 | 77164.3 | 6.33 | 66.22 | -0.429 | B3 | 121-222 |
|  |  |  |  |  |  |  |  |  | Auxin_resp | 290-373 |
|  |  | MdARF16 | XP_008366674.1 | 700 | 76872.34 | 6.35 | 68.41 | -0.406 | B3 | 121-222 |
|  |  |  |  |  |  |  |  |  | Auxin_resp | 290-373 |
|  |  | MdARF10 | NP_001280997.1 | 702 | 76890.14 | 6.45 | 68.22 | -0.398 | B3 | 121-222 |
|  |  |  |  |  |  |  |  |  | Auxin_resp | 290-373 |
|  |  | FvARF10 | XP_004304436.1 | 731 | 80694.48 | 7.34 | 68.17 | -0.44 | B3 | 149-250 |
|  |  |  |  |  |  |  |  |  | Auxin_resp | 318-401 |
|  |  | FvARF16 | XP_011468106.1 | 733 | 80884.7 | 7.33 | 67.98 | -0.436 | B3 | 149-250 |
|  |  |  |  |  |  |  |  |  | Auxin-resp | 318-401 |
|  |  | JrARF16 | XP_018838366.1 | 703 | 77417.74 | 6.3 | 70.9 | -0.385 | B3 | 121-222 |
|  |  |  |  |  |  |  |  |  | Auxin-resp | 292-375 |
|  |  |  |  |  |  |  |  |  | AUX_IAA | 595-697 |
|  |  | JrARF10 | XP_018838364.1 | 701 | 77026.5 | 6.27 | 69.83 | -0.324 | B3 | 121-222 |
|  |  |  |  |  |  |  |  |  | Auxin-resp | 292-375 |
|  |  |  |  |  |  |  |  |  | AUX_IAA | 619-701 |
|  |  | VvARF10 | VIT_208s0040g01810.1 | 701 | 77484.04 | 6.85 | 63.57 | -0.49 | B3 | 119-220 |
|  |  |  |  |  |  |  |  |  | Auxin_resp | 288-371 |
|  | Subgroup II | SlARF10 | XP_004246657.1 | 694 | 76222.22 | 6.64 | 71.77 | -0.403 | B3 | 121-222 |
|  |  |  |  |  |  |  |  |  | Auxin-resp | 287-370 |
|  |  |  |  |  |  |  |  |  | AUX_IAA | 610-692 |
|  |  | VvARF16 | VIT_213s0019g04380.1 | 683 | 75268.01 | 6.43 | 75.84 | -0.388 | B3 | 117-218 |
|  |  |  |  |  |  |  |  |  | Auxin-resp | 279-362 |
|  |  |  |  |  |  |  |  |  | AUX_IAA | 604-681 |
|  |  | AtARF16 | NP_567841.1 | 670 | 73978.8 | 6.95 | 74.34 | -0.409 | B3 | 120-221 |
|  |  |  |  |  |  |  |  |  | Auxin_resp | 279-362 |
|  |  | PaARF16 | XP_021832628.1 | 719 | 79093.28 | 7.88 | 72.43 | -0.41 | B3 | 112-213 |
|  |  |  |  |  |  |  |  |  | Auxin_resp | 286-369 |
|  |  | PmARF16 | XP_008245241.1 | 719 | 79276.48 | 8.31 | 70.13 | -0.429 | B3 | 112-213 |
|  |  |  |  |  |  |  |  |  | Auxin_resp | 286-369 |
|  |  | SlARF16 | NP_001234880.2 | 671 | 74911.05 | 5.69 | 79.12 | -0.272 | B3 | 103-204 |
|  |  |  |  |  |  |  |  |  | Auxin_resp | 262-345 |
|  |  | AtARF10 | NP_180402.1 | 693 | 76720.88 | 7.63 | 72.91 | -0.384 | B3 | 115-216 |
|  |  |  |  |  |  |  |  |  | Auxin-resp | 284-360 |
| Group II |  | AtARF17 | NP_565161.1 | 585 | 63741.97 | 5.5 | 64.32 | -0.445 | B3 | 119-219 |
|  |  |  |  |  |  |  |  |  | Auxin_resp | 274-356 |
|  |  | SlARF17 | NP_001234237.2 | 622 | 68392.16 | 5.58 | 71.25 | -0.321 | B3 | 110-213 |
|  |  |  |  |  |  |  |  |  | Auxin_resp | 263-340 |
|  |  | VvARF17 | VIT_218s0001g04180.1 | 550 | 60780.7 | 6 | 71.89 | -0.322 | B3 | 120-221 |
|  |  |  |  |  |  |  |  |  | Auxin_resp | 275-352 |
|  |  | JrARF17 | XP_018812230.1 | 596 | 65443.52 | 5.99 | 71.12 | -0.35 | B3 | 124-226 |
|  |  |  |  |  |  |  |  |  | Auxin_resp | 284-359 |
|  |  | FvARF17 | XP_011465821.1 | 510 | 56256.72 | 7.34 | 70.35 | -0.308 | B3 | 141-244 |
|  |  |  |  |  |  |  |  |  | Auxin_resp | 294-369 |
|  |  | MdARF17 | XP_008347772.1 | 593 | 65353.84 | 5.84 | 69.19 | -0.349 | B3 | 130-232 |
|  |  |  |  |  |  |  |  |  | Auxin_resp | 278-361 |
|  |  | PmARF17 | XP_008233939.1 | 603 | 66596.96 | 6.52 | 71.26 | -0.375 | B3 | 131-236 |
|  |  |  |  |  |  |  |  |  | Auxin_resp | 283-366 |
|  |  | PpARF17 | XP_007225689.1 | 600 | 66197.55 | 6.32 | 71.95 | -0.364 | B3 | 131-233 |
|  |  |  |  |  |  |  |  |  | Auxin_resp | 280-363 |
|  |  | PaARF17 | XP_021810562.1 | 600 | 66374.65 | 6.32 | 72.75 | -0.384 | B3 | 131-233 |
|  |  |  |  |  |  |  |  |  | Auxin_resp | 280-363 |

Noaa, Number of amino acids; pI, isoelectric point; Ai, aliphatic index; GRAVY, grand average of hydropathicity; MW, molecular weight.
