# Supplementary material for: VvmiR160s/VvARFs interaction and their spatio-temporal expression/cleavage products during GA-induced grape parthenocarpy
Source: BMC Plant Biol. 2019 Mar 21;19:111. doi: 10.1186/s12870-019-1719-9 (PMC6429806; doi:10.1186/s12870-019-1719-9)
Supplement: Supplementary file 4 — Table S2. All the motif information of VvMIR160s precursor genes and their targeted VvARF genes’ promoter.The Plantcare software (http://bioinformatics.psb.ugent.be/webtools/plantcare/html/) was used to predict the motif elements of these genes’ promoter. (DOCX 24 kb) [file 12870_2019_1719_MOESM4_ESM.docx]

**Additional file 4:**

**Table S2. All the motif information of VvmiR160s precursor genes and their targeted *VvARF* genes’ promoter.**

| Name | Elements | Sum | Number | Motif | Functions |
| --- | --- | --- | --- | --- | --- |
| VvmiR160a | light-related elements | 13 | 1 | ATCT-motif | part of a conserved DNA module involved in light responsiveness |
|  |  |  | 1 | Box 4 | part of a conserved DNA module involved in light responsiveness |
|  |  |  | 2 | Box I | light responsive element |
|  |  |  | 2 | G-box | cis-acting regulatory element involved in light responsiveness |
|  |  |  | 2 | GA-motif | part of a light responsive element |
|  |  |  | 2 | GAG-motif | part of a light responsive element |
|  |  |  | 1 | GATA-motif | part of a light responsive element |
|  |  |  | 1 | GT1-motif | light responsive element |
|  |  |  | 1 | MRE | MYB binding site involved in light responsiveness |
|  | hormone-related elements | 8 | 1 | ABRE | cis-acting element involved in the abscisic acid responsiveness |
|  |  |  | 1 | AuxRE | part of an auxin-responsive element |
|  |  |  | 1 | ERE | ethylene-responsive element |
|  |  |  | 2 | GARE-motif | gibberellin-responsive element |
|  |  |  | 1 | P-box | gibberellin-responsive element |
|  |  |  | 2 | TCA-element | cis-acting element involved in salicylic acid responsiveness |
|  | tissue specific elements | 5 | 2 | CAT-box | cis-acting regulatory element related to meristem expression |
|  |  |  | 1 | GCN4_motif | cis-regulatory element involved in endosperm expression |
|  |  |  | 2 | Skn-1_motif | cis-acting regulatory element required for endosperm expression |
|  | stress-related elements | 8 | 2 | ARE | cis-acting regulatory element essential for the anaerobic induction |
|  |  |  | 1 | Box-W1 | fungal elicitor responsive element |
|  |  |  | 4 | MBS | MYB binding site involved in drought-inducibility |
|  |  |  | 1 | TC-rich repeats | cis-acting element involved in defense and stress responsiveness |
|  | circadian | 1 | 1 | circadian | cis-acting regulatory element involved in circadian control |
| VvmiR160b | light-related elements | 15 | 4 | Box 4 | part of a conserved DNA module involved in light responsiveness |
|  |  |  | 1 | Box I | light responsive element |
|  |  |  | 2 | G-Box | cis-acting regulatory element involved in light responsiveness |
|  |  |  | 1 | GA-motif | part of a light responsive element |
|  |  |  | 2 | GAG-motif | part of a light responsive element |
|  |  |  | 1 | I-box | part of a light responsive element |
|  |  |  | 1 | LAMP-element | part of a light responsive element |
|  |  |  | 1 | Sp1 | light responsive element |
|  |  |  | 1 | TCT-motif | part of a light responsive element |
|  |  |  | 1 | chs-CMA2a | part of a light responsive element |
|  | tissue specific elements | 3 | 3 | Skn-1_motif | cis-acting regulatory element required for endosperm expression |
|  | stress | 10 | 3 | ARE | cis-acting regulatory element essential for the anaerobic induction |
|  |  |  | 1 | Box-W1 | fungal elicitor responsive element |
|  |  |  | 1 | GC-motif | enhancer-like element involved in anoxic specific inducibility |
|  |  |  | 1 | LTR | cis-acting element involved in low-temperature responsiveness |
|  |  |  | 3 | MBS | MYB binding site involved in drought-inducibility |
|  |  |  | 1 | TC-rich repeats | cis-acting element involved in defense and stress responsiveness |
|  | circadian | 1 | 1 | circadian | cis-acting regulatory element involved in circadian control |
| VvmiR160c | light-related elements | 24 | 1 | 3-AF1 binding site | light responsive element |
|  |  |  | 1 | ATCC-motif | part of a conserved DNA module involved in light responsiveness |
|  |  |  | 3 | Box I | light responsive element |
|  |  |  | 2 | CATT-motif | part of a light responsive element |
|  |  |  | 6 | G-Box | cis-acting regulatory element involved in light responsiveness |
|  |  |  | 2 | GA-motif | part of a light responsive element |
|  |  |  | 1 | GTGGC-motif | part of a light responsive element |
|  |  |  | 1 | I-box | part of a light responsive element |
|  |  |  | 1 | LAMP-element | part of a light responsive element |
|  |  |  | 3 | Sp1 | light responsive element |
|  |  |  | 2 | TCCC-motif | part of a light responsive element |
|  |  |  | 1 | rbcS-CMA7a | part of a light responsive element |
|  | hormone-related elements | 6 | 3 | ABRE | cis-acting element involved in the abscisic acid responsiveness |
|  |  |  | 1 | ERE | ethylene-responsive element |
|  |  |  | 1 | P-box | gibberellin-responsive element |
|  |  |  | 1 | TCA-element | cis-acting element involved in salicylic acid responsiveness |
|  | tissue specific elements | 3 | 3 | Skn-1_motif | cis-acting regulatory element required for endosperm expression |
|  | stress-related elements | 8 | 1 | ARE | cis-acting regulatory element essential for the anaerobic induction |
|  |  |  | 1 | Box-W1 | fungal elicitor responsive element |
|  |  |  | 5 | HSE | cis-acting element involved in heat stress responsiveness |
|  |  |  | 1 | MBS | MYB binding site involved in drought-inducibility |
| VvmiR160d | light-related elements | 23 | 3 | 5UTR Py-rich stretch | cis-acting element conferring high transcription levels |
|  |  |  | 2 | AE-box | part of a module for light response |
|  |  |  | 4 | Box I | light responsive element |
|  |  |  | 4 | G-box | cis-acting regulatory element involved in light responsiveness |
|  |  |  | 2 | GAG-motif | part of a light responsive element |
|  |  |  | 1 | GATA-motif | part of a light responsive element |
|  |  |  | 2 | I-box | part of a light responsive element |
|  |  |  | 1 | L-box | part of a light responsive element |
|  |  |  | 4 | Sp1 | light responsive element |
|  | hormone-related elements | 10 | 1 | CGTCA-motif | cis-acting regulatory element involved in the MeJA-responsiveness |
|  |  |  | 1 | ERE | ethylene-responsive element |
|  |  |  | 2 | GARE-motif | gibberellin-responsive element |
|  |  |  | 5 | TCA-element | cis-acting element involved in salicylic acid responsiveness |
|  |  |  | 1 | TGACG-motif | cis-acting regulatory element involved in the MeJA-responsiveness |
|  | tissue specific elements | 3 | 2 | CAT-box | cis-acting regulatory element related to meristem expression |
|  |  |  | 1 | GCN4_motif | cis-regulatory element involved in endosperm expression |
|  | stress-related elements | 4 | 1 | ARE | cis-acting regulatory element essential for the anaerobic induction |
|  |  |  | 1 | HSE | cis-acting element involved in heat stress responsiveness |
|  |  |  | 1 | MBS | MYB binding site involved in drought-inducibility |
|  |  |  | 1 | WUN-motif | wound-responsive element |
| VvmiR160e | light-related elements | 16 | 2 | 5UTR Py-rich stretch | cis-acting element conferring high transcription levels |
|  |  |  | 1 | Box 4 | part of a conserved DNA module involved in light responsiveness |
|  |  |  | 2 | Box I | light responsive element |
|  |  |  | 1 | G-box | cis-acting regulatory element involved in light responsiveness |
|  |  |  | 4 | GAG-motif | part of a light responsive element |
|  |  |  | 1 | I-box | part of a light responsive element |
|  |  |  | 2 | LAMP-element | part of a light responsive element |
|  |  |  | 2 | Sp1 | light responsive element |
|  |  |  | 1 | TCCC-motif | part of a light responsive element |
|  | hormone-related elements | 6 | 1 | CGTCA-motif | cis-acting regulatory element involved in the MeJA-responsiveness |
|  |  |  | 1 | ERE | ethylene-responsive element |
|  |  |  | 1 | TATC-box | cis-acting element involved in gibberellin-responsiveness |
|  |  |  | 2 | TCA-element | cis-acting element involved in salicylic acid responsiveness |
|  |  |  | 1 | TGACG-motif | cis-acting regulatory element involved in the MeJA-responsiveness |
|  | tissue specific elements | 2 | 1 | RY-element | cis-acting regulatory element involved in seed-specific regulation |
|  |  |  | 1 | Skn-1_motif | cis-acting regulatory element required for endosperm expression |
|  | stress-related elements | 6 | 3 | ARE | cis-acting regulatory element essential for the anaerobic induction |
|  |  |  | 1 | Box-W1 | fungal elicitor responsive element |
|  |  |  | 2 | TC-rich repeats | cis-acting element involved in defense and stress responsiveness |
| *VvARF16* | light-related elements | 23 | 1 | AE-box | part of a module for light response |
|  |  |  | 7 | Box 4 | part of a conserved DNA module involved in light responsiveness |
|  |  |  | 1 | Box I | light responsive element |
|  |  |  | 1 | CATT-motif | part of a light responsive element |
|  |  |  | 4 | G-Box | cis-acting regulatory element involved in light responsiveness |
|  |  |  | 6 | GT1-motif | light responsive element |
|  |  |  | 1 | I-box | light responsive element |
|  |  |  | 2 | Sp1 | light responsive element |
|  | hormone-related elements | 6 | 1 | AuxRR-core | cis-acting regulatory element involved in auxin responsiveness |
|  |  |  | 1 | CGTCA-motif | cis-acting regulatory element involved in the MeJA-responsiveness |
|  |  |  | 1 | ERE | ethylene-responsive element |
|  |  |  | 1 | P-box | gibberellin-responsive element |
|  |  |  | 1 | TCA-element | cis-acting element involved in salicylic acid responsiveness |
|  |  |  | 1 | TGACG-motif | cis-acting regulatory element involved in the MeJA-responsiveness |
|  | tissue specific elements | 6 | 6 | Skn-1_motif | cis-acting regulatory element required for endosperm expression |
|  | stress-related elements | 11 | 3 | ARE | cis-acting regulatory element essential for the anaerobic induction |
|  |  |  | 4 | HSE | cis-acting element involved in heat stress responsiveness |
|  |  |  | 2 | LTR | cis-acting element involved in low-temperature responsiveness |
|  |  |  | 2 | MBS | MYB binding site involved in drought-inducibility |
|  | circadian | 1 | 1 | circadian | cis-acting regulatory element involved in circadian control |
| *VvARF10* | light-related elements | 25 | 2 | ACE | cis-acting element involved in light responsiveness |
|  |  |  | 1 | AE-box | part of a module for light response |
|  |  |  | 2 | ATCT-motif | part of a conserved DNA module involved in light responsiveness |
|  |  |  | 3 | Box 4 | part of a conserved DNA module involved in light responsiveness |
|  |  |  | 5 | Box I | light responsive element |
|  |  |  | 1 | CATT-motif | part of a light responsive element |
|  |  |  | 4 | G-Box | cis-acting regulatory element involved in light responsiveness |
|  |  |  | 2 | GAG-motif | part of a light responsive element |
|  |  |  | 4 | GT1-motif | light responsive element |
|  |  |  | 1 | TCT-motif | part of a light responsive element |
|  | hormone-related elements | 5 | 1 | ABRE | cis-acting element involved in the abscisic acid responsiveness |
|  |  |  | 1 | ERE | ethylene-responsive element |
|  |  |  | 1 | P-box | gibberellin-responsive element |
|  |  |  | 2 | TCA-element | cis-acting element involved in salicylic acid responsiveness |
|  | tissue specific elements | 3 | 1 | CAT-box | cis-acting regulatory element related to meristem expression |
|  |  |  | 1 | GCN4_motif | cis-regulatory element involved in endosperm expression |
|  |  |  | 1 | Skn-1_motif | cis-acting regulatory element required for endosperm expression |
|  | stress-related elements | 9 | 3 | ARE | cis-acting regulatory element essential for the anaerobic induction |
|  |  |  | 1 | Box-W1 | fungal elicitor responsive element |
|  |  |  | 2 | MBS | MYB binding site involved in drought-inducibility |
|  |  |  | 3 | TC-rich repeats | cis-acting element involved in defense and stress responsiveness |
|  | circadian | 1 | 1 | circadian | cis-acting regulatory element involved in circadian control |
| *VvARF17* | light-related elements | 15 | 2 | AE-box | part of a module for light response |
|  |  |  | 1 | AT1-motif | part of a light responsive module |
|  |  |  | 1 | ATCT-motif | part of a conserved DNA module involved in light responsiveness |
|  |  |  | 3 | Box 4 | part of a conserved DNA module involved in light responsiveness |
|  |  |  | 2 | Box I | light responsive element |
|  |  |  | 1 | GT1-motif | light responsive element |
|  |  |  | 1 | MNF1 | light responsive element |
|  |  |  | 1 | MRE | MYB binding site involved in light responsiveness |
|  |  |  | 2 | TCCC-motif | part of a light responsive element |
|  |  |  | 1 | TCT-motif | part of a light responsive element |
|  | hormone-related elements | 7 | 2 | CGTCA-motif | cis-acting regulatory element involved in the MeJA-responsiveness |
|  |  |  | 2 | GARE-motif | gibberellin-responsive element |
|  |  |  | 1 | TCA-element | cis-acting element involved in salicylic acid responsiveness |
|  |  |  | 2 | TGACG-motif | cis-acting regulatory element involved in the MeJA-responsiveness |
|  | tissue specific elements | 3 | 3 | Skn-1_motif | cis-acting regulatory element required for endosperm expression |
|  | stress-related elements | 7 | 5 | HSE | cis-acting element involved in heat stress responsiveness |
|  |  |  | 1 | LTR | cis-acting element involved in low-temperature responsiveness |
|  |  |  | 1 | MBS | MYB binding site involved in drought-inducibility |
|  | circadian | 1 | 1 | circadian | cis-acting regulatory element involved in circadian control |

Note: The Plantcare software (http://bioinformatics.psb.ugent.be/webtools/plantcare/html/) was used to predict the motif elements of these genes’ promoter.
