# Supplementary material for: VvmiR160s/VvARFs interaction and their spatio-temporal expression/cleavage products during GA-induced grape parthenocarpy
Source: BMC Plant Biol. 2019 Mar 21;19:111. doi: 10.1186/s12870-019-1719-9 (PMC6429806; doi:10.1186/s12870-019-1719-9)
Supplement: Supplementary file 5 — Table S3. Motif elements related to hormone signals of MIR160s precursors’ genes and their targeted ARFs pairs in grapevine. The Plantcare software (http://bioinformatics.psb.ugent.be/webtools/plantcare/html/) was used to predict the motif elements of these genes’ promoter. (DOCX 16 kb) [file 12870_2019_1719_MOESM5_ESM.docx]

**Additional file 5: Table S3. Motif elements related to hormone signals of miR160s precursors’ genes and their targeted *ARFs* pairs in grapevine.**

| Name | Sum | Number | Elements | Functions |
| --- | --- | --- | --- | --- |
| VvmiR160a | 8 | 1 | ABRE | cis-acting element involved in the abscisic acid responsiveness |
|  |  | 1 | AuxRE | part of an auxin-responsive element |
|  |  | 1 | ERE | ethylene-responsive element |
|  |  | 2 | GARE-motif | gibberellin-responsive element |
|  |  | 1 | P-box | gibberellin-responsive element |
|  |  | 2 | TCA-element | cis-acting element involved in salicylic acid responsiveness |
| VvmiR160b | 0 | 0 |  |  |
| VvmiR160c | 6 | 3 | ABRE | cis-acting element involved in the abscisic acid responsiveness |
|  |  | 1 | ERE | ethylene-responsive element |
|  |  | 1 | P-box | gibberellin-responsive element |
|  |  | 1 | TCA-element | cis-acting element involved in salicylic acid responsiveness |
| VvmiR160d | 10 | 1 | CGTCA-motif | cis-acting regulatory element involved in the MeJA-responsiveness |
|  |  | 1 | ERE | ethylene-responsive element |
|  |  | 2 | GARE-motif | gibberellin-responsive element |
|  |  | 5 | TCA-element | cis-acting element involved in salicylic acid responsiveness |
|  |  | 1 | TGACG-motif | cis-acting regulatory element involved in the MeJA-responsiveness |
| VvmiR160e | 6 | 1 | CGTCA-motif | cis-acting regulatory element involved in the MeJA-responsiveness |
|  |  | 1 | ERE | ethylene-responsive element |
|  |  | 1 | TATC-box | cis-acting element involved in gibberellin-responsiveness |
|  |  | 2 | TCA-element | cis-acting element involved in salicylic acid responsiveness |
|  |  | 1 | TGACG-motif | cis-acting regulatory element involved in the MeJA-responsiveness |
| *VvARF16* | 6 | 1 | AuxRR-core | cis-acting regulatory element involved in auxin responsiveness |
|  |  | 1 | CGTCA-motif | cis-acting regulatory element involved in the MeJA-responsiveness |
|  |  | 1 | ERE | ethylene-responsive element |
|  |  | 1 | P-box | gibberellin-responsive element |
|  |  | 1 | TCA-element | cis-acting element involved in salicylic acid responsiveness |
|  |  | 1 | TGACG-motif | cis-acting regulatory element involved in the MeJA-responsiveness |
| *VvARF10* | 5 | 1 | ABRE | cis-acting element involved in the abscisic acid responsiveness |
|  |  | 1 | ERE | ethylene-responsive element |
|  |  | 1 | P-box | gibberellin-responsive element |
|  |  | 2 | TCA-element | cis-acting element involved in salicylic acid responsiveness |
| *VvARF17* | 7 | 2 | CGTCA-motif | cis-acting regulatory element involved in the MeJA-responsiveness |
|  |  | 2 | GARE-motif | gibberellin-responsive element |
|  |  | 1 | TCA-element | cis-acting element involved in salicylic acid responsiveness |
|  |  | 2 | TGACG-motif | cis-acting regulatory element involved in the MeJA-responsiveness |

Note: The Plantcare software (http://bioinformatics.psb.ugent.be/webtools/plantcare/html/) was used to predict the motif elements of these genes’ promoter.
