# Supplementary material for: VvmiR160s/VvARFs interaction and their spatio-temporal expression/cleavage products during GA-induced grape parthenocarpy
Source: BMC Plant Biol. 2019 Mar 21;19:111. doi: 10.1186/s12870-019-1719-9 (PMC6429806; doi:10.1186/s12870-019-1719-9)
Supplement: Supplementary file 6 — Table S4. List of the primers used for experiments. (DOCX 16 kb) [file 12870_2019_1719_MOESM6_ESM.docx]

**Additional file 6: Table S4**. **Primers used for experiments.**

| Name | Forward primer sequences (5’-3’) | Reverse primer sequences (5’-3’) | Use |
| --- | --- | --- | --- |
| P1 | ATGATTCCATTTTTGGGTTCAAA | TTATTTTCTATACACTCCTACATTGTC | Amplification of *VvARF10* ORF |
| P2 | ATGATCAGTCTTATGGATCCCAT | CTAGGGTGTTGCTGCCGAA | Amplification of *VvARF16* ORF |
| P3 | ATGTGTCCCCTCCCGGCG | TCATTGTATTTTTTTACTACCATCTTC | Amplification of *VvARF17* ORF |
| P4 | GTTCCAGTTAACCTCTACCTTCTCG | TTCAATCAATGTTGTGATAGTAATCA | Amplification of *VvARF10* promoter 1 |
| P5 | CATTGAATGAATCTTTGAAAACTTTTA | TTCAATCAATGTTGTGATAGTAATCAA | Amplification of *VvARF10* promoter 2 |
| P6 | AGACATGACTGATATTTTTTTTATAGG | CACCTTTCTGTTCTGTTCTGTTC | Amplification of *VvmiR160c* promoter 1 |
| P7 | AGACATGACTGATATTTTTTTTATAGG | GAGCTACCACCAAAATCCTCTC | Amplification of *VvmiR160c* promoter 2 |
| P8 | TGTGACATGAATATGGTGCG | CACCTCCTAAAATCATTGTCTG | Amplification of the primary transcripts of VvmiR160a |
| P9 | TCTGATTGAATTACATGAAGGAGGT | ATAAATTGGAAAATGAATGGTCACA | Amplification of the primary transcripts of VvmiR160b |
| P10 | CTCTGGCCTGGCCTCTATAAAT | GCACAACCCATTTCACCTTT | Amplification of the primary transcripts of VvmiR160c |
| P11 | CAGGAAATTTATTCTAATACCACTA | CTAACAAGGAAGAAAATGTG | Amplification of the primary transcripts of VvmiR160d |
| P12 | CCTCCTCCTCTCCTCCTCCT | GAGAAACATCACATCACCAAAAC | Amplification of the primary transcripts of VvmiR160e |
| P13 | GCTATCTGGGTTGACCTCCA | TGACCTTTGTGCTTCAGTGG | Real time RT-PCR for VvmiR160a |
| P14 | TTCTGCAGGAGATGGAGCTT | AGTGTTTCGCCTGCTTGACT | Real time RT-PCR for VvmiR160b |
| P15 | CCACATTCCGTGACCTTTCT | TGGAGCTACCACCAAAATCC | Real time RT-PCR for VvmiR160c |
| P16 | TGCTTAACCATTCCCTCTCC | GCTTTGTGCATGTGGGAGTA | Real time RT-PCR for VvmiR160d |
| P17 | AGGGTATTTCCGCCATTTTC | GGGGGAGAAGATTGAAGAGG | Real time RT-PCR for VvmiR160e |
| P18 | TGCAGTCAAACATATCGGCG | CACTCCTACATTGTCGCTGC | Real time RT-PCR for *VvARF10* |
| P19 | GGTCGTCTACTTCCCTCAGG | CTGGTTTCTCAGGAGCCTCA | Real time RT-PCR for *VvARF16* |
| P20 | GAAGCGCTGAGTGTCTTCTG | CCTCCAAGGCCCATTATCCA | Real time RT-PCR for *VvARF17* |
| P21 | TACAATTCCATCATGAAGTGTGATG | TTAGAAGCACTTCCTGTGAACAATG | *Actin* primer |
